# SIMPLEX: Cloud-Enabled Pipeline for the Comprehensive Analysis of Exome Sequencing Data

**DOI:** 10.1371/journal.pone.0041948

**Published:** 2012-08-01

**Authors:** Maria Fischer, Rene Snajder, Stephan Pabinger, Andreas Dander, Anna Schossig, Johannes Zschocke, Zlatko Trajanoski, Gernot Stocker

**Affiliations:** 1 Division for Bioinformatics, Biocenter, Innsbruck Medical University, Innsbruck, Austria; 2 Oncotyrol, Center for Personalized Cancer Medicine, Innsbruck, Austria; 3 Division of Human Genetics, Biocenter, Innsbruck Medical University, Innsbruck, Austria; CSIR-Institute of Microbial Technology, India

## Abstract

In recent studies, exome sequencing has proven to be a successful screening tool for the identification of candidate genes causing rare genetic diseases. Although underlying targeted sequencing methods are well established, necessary data handling and focused, structured analysis still remain demanding tasks. Here, we present a cloud-enabled autonomous analysis pipeline, which comprises the complete exome analysis workflow. The pipeline combines several in-house developed and published applications to perform the following steps: (a) initial quality control, (b) intelligent data filtering and pre-processing, (c) sequence alignment to a reference genome, (d) SNP and DIP detection, (e) functional annotation of variants using different approaches, and (f) detailed report generation during various stages of the workflow. The pipeline connects the selected analysis steps, exposes all available parameters for customized usage, performs required data handling, and distributes computationally expensive tasks either on a dedicated high-performance computing infrastructure or on the Amazon cloud environment (EC2). The presented application has already been used in several research projects including studies to elucidate the role of rare genetic diseases. The pipeline is continuously tested and is publicly available under the GPL as a VirtualBox or Cloud image at http://simplex.i-med.ac.at; additional supplementary data is provided at http://www.icbi.at/exome.

## Introduction

During the last years, the rapid development of next generation sequencing (NGS) technologies [1]–[3] sustainably extended the possibilities of scientific work in biology and medicine but, at the same time, confronted researchers with an overwhelming flood of data. Different sequencing platforms became available (e.g. Roche 454 FLX [4], Illumina Genome Analyzer [5], and SOLiD system) and increased the discrepancy of being able to generate sequence data and extracting relevant information out of performed experiments even more. Hand in hand with the increase of sequencing throughput, the cost per base dropped from $10 in 1985 [6] to fractions of cents in 2011 [7] and made large scale studies including whole-genome approaches affordable.

Although the costs of human whole-genome sequencing dropped significantly, it still remains a time consuming and expensive method. Therefore, exome sequencing proved to be a valuable alternative for the investigation of several diseases including rare Mendelian disorders [8], [9]. In this approach, only the protein coding regions of the DNA are sequenced, which contain around 180,000 exons and form approximately one percent of the human genome [10]. Exome sequencing studies primarily aim at the discovery of single nucleotide polymorphisms (SNPs) and deletion/insertion polymorphisms (DIPs) to identify disease-causing variants in clinical samples, as it is assumed that about 85% of these mutations can be found in protein coding regions [11].

The current bottleneck of NGS projects is not mainly the sequencing of DNA itself, but lies in the structured way of data management and the targeted computational analysis of experiments [12]. The coordinated, systematic storage and backup of data is still challenging to most laboratories, and many dedicated analysis methods require deep methodological knowledge and a powerful computational infrastructure. Recently, the introduction of cloud computing has created new possibilities to analyze NGS data at reasonable costs [12], [13], especially for laboratories lacking a dedicated bioinformatics infrastructure. Still, the facilitation of exome-seq analysis by developing comprehensive and intuitive software suites is one of the main goals of bioinformaticians working with NGS data.

To this end, several NGS analysis pipelines were published [14]–[19] that are suitable for the investigation of exome-seq data. However, they either do not cover the complete analysis workflow or require the fulfillment of cumbersome and tediousness prerequisites. Especially the installation and configuration of analysis tools and databases are challenging tasks for most inexperienced users. Moreover, based on the diversity and the lack of standards for NGS analysis, many different tools and data formats were introduced, posing a problem when combining different methods to conclude the analysis and obtain biological meaningful results [12]. The selection of adequate tools, applying appropriate parameters, and combining them to a streamlined analysis pipeline is a challenge which is often underestimated and requires advanced bioinformatics skills.

To overcome these challenges, an automatized pipeline, called SIMPLEX, for analyzing exome sequencing data has been developed. The pipeline is able to handle single-end (SE) as well as paired-end (PE) data and is able to process input data encoded in nucleotide space or color space. SIMPLEX combines published and in-house developed applications and is continuously, automatically tested. To facilitate the analysis of exome-seq data, especially in small scale laboratories, the pipeline is offered as a fully functional VirtualBox image that requires no additional installation of tools and databases. Furthermore, the pipeline provides a transparent usage of the high performance computing (HPC) infrastructure and offers quick access through a dedicated Cloud image.

## Methods

### Pipeline Overview

**Figure 1:**
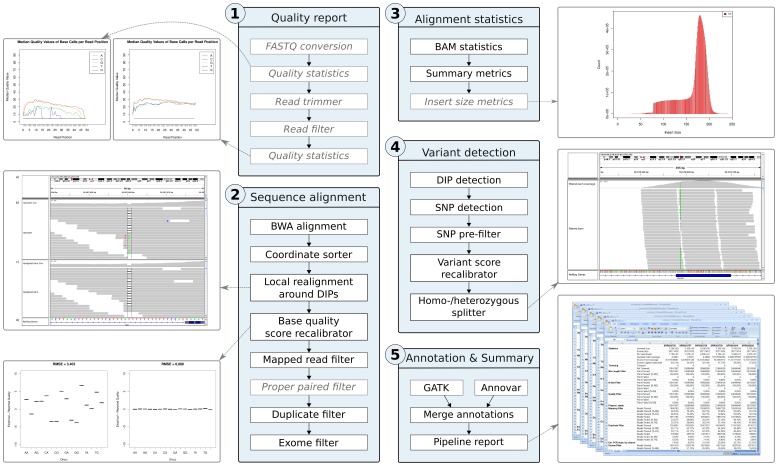
Schematic overview. The SIMPLEX analysis pipeline contains five major steps (blue boxes), which are further divided into several components. Mandatory components are depicted in black, optional in gray. The first step of the pipeline includes calculations of quality statistics on raw and processed reads, and applies filters and trimmers on sequenced reads (quality report). Afterwards, the pipeline aligns the processed reads to a reference genome (sequence alignment), performs alignment statistics and region filtering (alignment statistics), and detects variants resulting in a list of potential disease driver candidates (variant detection). Output files can be visualized using standard genome viewers. At the end, the pipeline automatically annotates variants, generates a detailed summary report, and combines calculated results including key figures in a structured way (annotation & summary).

SIMPLEX is a comprehensive pipeline for investigating exome SE and PE sequencing data generated by deep sequencing devices from Illumina and ABI SOLiD. It exposes a wide variety of parameters to offer great flexibility for analyzing data according to the given biological problem and, at the same time, provides a well chosen set of standard parameters for unversed users. SIMPLEX requires as input the raw sequence reads, their corresponding base calling quality values, and a list of genomic positions specifying the complete exome. A default exome pipeline analysis with SIMPLEX includes all steps depicted in Figure 1 and is elaborated in the following.

### Input Files

The pipeline is able to handle different input file formats which result from combining specific library preparation protocols with various sequencing platforms. Data produced by Illumina devices need to be in FASTQ file format (Sanger, Solexa, or Illumina 1.3+, see [20]) whereas ABI SOLiD data require to be given in two separate files - csfasta and qual files, both in FASTA format. For all platforms PE information must be given by adding additional files representing the second reads in pair.

### Read Quality Control and Preprocessing

This part of the pipeline generates a basic overview on raw sequence reads, handles conversion to standardized file formats, and enhances the overall read quality by sophisticated filtering and read trimming. All analysis steps conducted within this component are highly customizable to meet the needs of different sequencing devices and library preparation methods. An overview is shown in the first step of Figure 1.

The first component handles either the conversion of Solexa and Illumina 1.3+ FASTQ format into Sanger FASTQ or the preparation of ABI SOLiD data to be readable by the sequence aligner. Next, read characteristics and read quality characteristics are calculated and exported as PDF report. Amongst other information, the report depicts the read length distribution, base call and base call quality distribution, and characteristics of unidentified base calls.

The read trimmer step is used to truncate FASTQ entries based on a given read length, nucleotide, or quality value. Furthermore, read filters can be applied to eliminate short or error prone sequence reads. The pipeline offers a *length filter*, a *quality filter*, and an *unidentified read call filter*, which can be applied sequentially on the provided data.

After filtering and trimming, quality statistics are created once more, which allow researchers to get a complete, appealing overview of performed read quality improvements.

### Sequence Alignment and Refinement

After the reads were preprocessed and low quality reads were filtered out, the sequence alignment software BWA [21] individually aligns the remaining reads to the chosen reference genome (see step two of Figure 1). Before executing the alignment process, it is important to consider the characteristics of the sequencing platform (nucleotide or color space) because specific alignment indices for the reference genome are required [22]. However, the indexing has to be performed only once for each reference genome and hereby generated indices are already included in the pipeline for widely used organisms.

#### Multiple local realignment around mutations

The initial alignment of sequence reads includes alignment artifacts due to the suboptimal characteristics of single alignment algorithms. Therefore, multiple local realignment around putative deletions and insertions (DIPs) is necessary to correct for alignment artifacts by minimizing the number of mismatching bases across all reads. SIMPLEX uses the realignment algorithm of the Genome Analysis Toolkit (GATK) [15], which has been optimized in-house to analyze reads in parallel. Since multiple local realignment is very time consuming, only sites likely requiring a realignment are processed.

#### Base quality recalibration

Systematic bias introduced by the initial base calling quality calculations are corrected using the base quality score recalibrator of GATK. It corrects the co-variation of the assigned quality value considering (i) the position within the sequence read, (ii) preceding and current nucleotide calls, and (iii) the probability of mismatching the reference genome. After performing base quality recalibration, the pipeline creates summarizing reports of this step.

#### Alignment filtering

Using the improved quality values, a critical filtering step removes unmapped and improperly paired reads. Furthermore, it detects unwanted PCR-duplicates and excludes reads which do not overlap with exonic regions of the reference genome. These filters can be fine-tuned by setting individual parameters.

### Alignment Statistics

The third step (see Figure 1) calculates several analysis statistics that are useful for evaluating data quality and alignment results before performing variant detection. All parts of this section are applied on reads which passed all precedent filters. The *BAM statistics* module provides a quick summary of the performed alignment, including total number of reads, number of mapped and unmapped reads, and read coverage in relation to the genome size. *Alignment summary metrics* report high level metrics about the alignment, such as median read length, deletions/insertions rate, and number of bases of high quality aligned reads. Furthermore, the pipeline reports *insert size metrics* that are useful to evaluate the insert size distribution of PE data.

### Variant Detection

The next analysis component (step four in Figure 1) deals with the identification of variants and is aimed at refining all variant calls to improve accuracy. In order to facilitate the search for recessive or dominant causes, variants are divided into homo- and heterozygous mutations. DIP calling, SNP identification, and variant score recalibration are carried out by GATK.

#### DIP caller

DIPs are detected by combining multiple sources, such as the number of reads covering a DIP site, read mapping qualities, and mismatch counts. Next, this initial set of DIP calls is filtered to remove false positives. The generated results are reported as Variant Call Format (VCF)-, as text (TXT)-, and as Browser Extensible Data (BED) files, which can be displayed in Genome Browser tracks. In order to accelerate DIP identification, the pipeline evenly divides the input data and executes DIP calling in parallel.

#### SNP caller

A raw set of SNP calls is determined by comparing the reference genome with the consensus sequence, which was previously deferred from the read alignment information by a Bayesian identifier. To improve runtime performance, SNP positions close to previously identified DIPs are ignored during sequence comparison. The identified SNPs are reported in VCF file format and are separated in homozygous and heterozygous variants. The subsequent SNP filter masks ambiguous SNP calls to create an improved SNP call set. This set is used as training data for variant score recalibration, which aims at improving the biological variant estimation.

### Mutation Annotation

Variant annotation is a two-step process, that comprises (1) adding information to already known mutations and (2) providing de- novo information for unknown variants.

The first component (see step five in Figure 1) uses the annotation function of GATK to add annotation information from existing databases and public resources. Amongst others, variants are annotated with RefSeq name [23], RefSeq hyperlink, GO term [24], KEGG [25], and dbSNP [26] information.

The second component applies the summarize_annovar function of ANNOVAR [27] on all variant files. In addition to adding information for known mutations (e.g.: allele frequencies as determined by the 1000 genomes project), the method uses inheritance models to deduce the exonic function of unknown variants. Furthermore, it reports normalized scores for identified variants from numerous tools (SIFT, PolyPhen2, PhyloP, MutationTaster, LRT), which try to predict the severity of mutations.

Finally, results from both annotation components are merged together into a structured and easily readable, tab delimited file.

### Pipeline Report Generation

The last component of the pipeline collects summary information from log and result files generated during the pipeline run and outputs the report as an MS-Excel file (see step five of Figure 1). The first section contains informative key figures regarding the alignment including *exome size*, *genome and exome fold coverage*, *exome capture specificity*, and *estimated PCR duplicates by chance*.

Next, the performance of all applied filters is reported including percentage values of passed and filtered reads. Summarizing information about detected DIPs contains figures such as the number of deletions, insertions, DIPs associated with RefSeq, and the number of DIPs in coding regions. The final section summarizes information about all identified SNPs.

### Pipeline Architecture

The methodological basis of the pipeline consists of a Java Enterprise Edition web service (JClusterService), which forms a dynamically extensible calculation back end and provides all necessary functionality to the pipeline users. This in-house developed API allows the delegation of numerical intensive calculations to a HPC infrastructure, which can be located in a dedicated server room or in a public cloud environment.

The pipeline client, which can be started on a regular office PC, transfers the raw sequencing data over secure web access to the calculation back-end and coordinates the parallel execution of analysis steps. Already completed result files are transferred back to the client workstation, and available summary information is collected throughout the complete pipeline run. The client side can be terminated during execution and easily restarted with the same parameters in order to continue the previously interrupted run. The service allows multiple users to perform multiple analyses at the same time and secures access to data by a central usermanagement system.

The client needs an adequate network connection to the server which is hosting JClusterService and an installed Java Runtime Environment. Since the result files of the pipeline can be several gigabytes, e.g. if mapped reads are fetched from the calculation back-end, increased storage capacity on the client side might be an additional requirement.

### Cloud Computing

A cloud image including SIMPLEX and all required programs has been created within Amazon’s Elastic Compute Cloud (EC2). This way, anyone with an Amazon EC2 account can instantiate the image with little to no effort and can run SIMPLEX without local compute facilities or advanced technical know-how. The EC2 cloud instantaneously provides the pipeline users with the full set of functions required to analyze exome sequencing data.

The cloud image is based on CloudBioLinux [28] and uses Galaxy CloudMan [29] to offer a straightforward and secured webinterface for the configuration and dynamic allocation of resources. Additional compute nodes or storage space can easily be added, and SIMPLEX can immediately make full use of the provided resources.

Once started, the user has the choice to either transfer the data manually to the cloud storage to analyze it with SIMPLEX, or call the pipeline client from a local machine, which will then automatically transfer the raw data to the cloud image and fetch the desired results.

### Application Usage

The pipeline is currently controlled through a command line interface, where input files and pipeline parameters can be specified. Although only a few parameters are required to run SIMPLEX, there is a large number of optional parameters that allow in- depth customization for specific biological questions (Table 1 and Table S1 provide a list of mandatory and all available parameters, respectively).

**Table 1:** Mandatory pipeline parameters.

Parameter	Name	Description
-c	command	which pipeline should be run (*exomeSE* or *exomePE*)
-od	output directory	directory where result files are stored
-genP	genome prefix	prefix of the reference genome (e.g. hg18, hg19)
-I	input files	list of files containing raw sequence reads^1^
-sfeb	SAM exome option	bed file determining the exone regions
-dsP	dip splitter option	percentage to distinguish between homo- and heterozygous DIPs
-k	Cluster profile	configuration file to access the cluster service

Listed are all parameters that need to be specified when starting the pipeline.

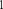
If PE data is given, the file names need to end with _R1 or _R2.

The complete software installation can be downloaded as a VirtualBox image or is available as EC2 Cloud image. No further installation is required. The VirtualBox image can be run on any system that has at least 4 CPU cores and more than 4GB of main memory. To achive a reasonable performance, an installation on an HPC infrastructure or (if the former is not available) the use of the EC2 Cloud image is recommended.

A detailed user manual is available in the supplementary section (see Supplementary Material S1), where all functions and parameters are explained, including how to use the SIMPLEX VirtualBox and Cloud image.

## Results

The presented autonomous pipeline for investigating exome sequencing data, SIMPLEX, allows researchers to analyze data generated by Illumina and ABI SOLiD NGS devices. It supports SE and PE data and takes advantage of HPC infrastructures or the EC2 Cloud to perform intensive calculations in a timely manner. The pipeline requires sequence reads, their corresponding base calling quality values, and a list of exon positions specifying the complete exome as input. The manifold results of the pipeline include a detailed summary report, files that can be used for viewing mapping results in Genome Browsers, and annotated lists of variants, which can be easily opened with office programs (see Table 2).

**Table 2:** Description of output files.

Name	Format	Description
read qualities	pdf	read quality statistics report available for raw and refined reads
read alignment	bam, bai	result files of alignment and alignment filtering steps
insert size distribution	png	insert size histogram (provided for PE data only)
exon counts	tsv	number of covering reads and fold coverage per exon
mutations	vcf, tsv	list of detected mutations
summary report	tsv, xlsx	detailed report of the analysis including several key figures

Listed are key intermediate and final results that are created by the pipeline.

### Evaluation of the Pipeline

To assess the performance on real biological data, we downloaded raw sequencing data generated by a study of the Kabuki syndrome [9] from the Sequence Read Archive (phs000295.v1.p1). The study comprised 42 runs, both SE and PE, from 10 different patients produced by Illumina Genome Analyzer II. All reads were mapped to the human genome primary assembly from the Genome Reference Consortium GRCh37NCBI (hg19). The results of the evaluation are described below and summarized in Table 3.

**Table 3:** Detailed results of SIMPLEX evaluation.

	SE Samples	PE samples
reads passed preprocessing	98%	100%
reads mappable	63%	54%
reads used for variant detection	23%	16%
number of raw SNPs	14,926	17,858
number of filtered SNPs	6,357	7,875
number of DIPs	473	402
raw SNPs in dbSNP	92%	76%
filtered SNPs in dbSNP	99%	98%
DIPs with RefSeq association	99%	99%
raw loss-of-function SNPs	1,181	4,098
filtered loss-of-function SNPs	593	1329
loss-of-function DIPs	47	96
missense/nonsens raw SNPs	737	1,562
missense/nonsens filtered SNPs	497	1,098

Listed are key figures (in avg.) for SE and PE samples.

A performance summary of the pipeline is depicted in Table 4, listing descriptive values for the runtime for SE and PE analysis runs. All samples were analyzed in parallel on a HPC infrastructure with 128 cores and 1 TB of memory.

**Table 4:** Runtime summary for Kabuki syndrome study.

Statistic	SE Samples	PE samples
mean pipeline runtime	12∶39	21∶19
median pipeline runtime	12∶54	24∶59
longest pipeline runtime	15∶14	26∶37
shortest pipeline runtime	07∶39	05∶13
mean longest step (local realignment)	02∶45	06∶59
mean alignment duration	02∶50	04∶52
mean SNP calling duration	00∶05	00∶06

Listed are overall and key runtime statistics (in hours).

#### Read preprocessing

Initial read preprocessing included the components *read-trimming* (truncating 5′ and 3′ Ns of a read, and removing base calls flagged by the Read Segment Quality Control Indicator), *min-length-filter* (filters out reads shorter than 25), *n-max-filter* (removes reads with more than five percent unidentified base calls), and *quality-filter* (filters out reads with more than five percent unreliable base calls). On average, 98% of SE and 100% of PE reads passed all preprocessing steps, indicating that raw files had already been preprocessed before publication (see Supplementary Material S2 for detailed results).

#### Alignment

BWA was started with the default parameter set followed by several filters. On average, 63% of SE and 54% PE reads could be mapped to the human genome. PE reads were subsequently applied to a proper-paired-filter where 79% of the reads passed the filter. Duplicate filtering removed 12% of SE and 4% of PE reads. After all exome-filtering steps, 23% of SE and 16% of PE *raw* reads remained for variant detection.

#### Variant detection

Variant detection identified on average 14,926 raw SNPs, 6,357 filtered SNPs, and 471 DIPs for SE, as well as 17,858 raw SNPs, 7,875 filtered SNPs, and 402 DIPs for PE reads. The calculated transition-transversion ratio for SNPs ranged from 2.15 to 3.58 with an average of 3.32.

#### Mutation annotation

Variants were annotated with additional information using the GATK annotator and the ANNOVAR software. On average 83% of all SNPs were referenced to dbSNP and 99% of all DIPs had an association to the RefSeq database. Furthermore, we identified 64,670 unique loss-of-function (nonsynonymous or frameshift) mutations in all investigated samples. Moreover, 24,093 variants were annotated as either missense or nonsense mutations.

#### Pipeline report generation

All automatically generated summary reports were combined into a single document (see Table S2). Amongst other key figures reported, the exome capture specificity was 37.7% for SE reads and 30.2% for PE reads. Exome fold coverage was reported to be 13.2 fold for SE and 17.5 fold for PE. Moreover, we identified that 18.8% of exons (as specified by CCDS [30]) were not covered (<1x) by SE runs, whereas around 7% had a coverage above 20 fold (SE). PE runs did on average not cover 25% of the exome, but approximately 21% were highly covered.

**Table 5:** Comparison of exome analysis tools.

Criteria	SIMPLEX	ngs– backbone*^a^*	GATK*^b^*	inGAP*^c^*	SeqGene*^d^*	GAMES*^e^*	TREAT*^f^*	Atlas2*^g^*
Free of charge	✓	✓	✓	✓	✓	✓	✓	✓
SE/PE data handling	✓/✓	✓/−	✓/✓	✓/✓	n.m.	✓/✓	✓/✓	✓/✓
NS/CS data handling	✓/✓	✓/✓	✓/✓	✓/−	n.m.	✓/✓	✓/−	✓/✓
Alignment	✓	✓	-	✓	✓	-	✓	-
Variant annotation	✓	-	✓	-	✓	✓	✓	-
Highly customizable	✓	✓	✓	-	✓	✓	✓	-
PCR duplicate handling	✓	-	-	-	-	✓	-	-
Homo−/heterozygosity	✓/✓	−/−	✓/✓	−/−	✓/✓	−/−	✓/✓	✓/✓
Quality reports	✓	✓	✓	-	✓	-	✓	-
Summary reports	✓	-	-	✓	-	✓	-	-
HPC support	✓	✓	✓	-	-	-	✓	-
Cloud support	✓	-	-	-	-	-	✓	✓
Graphical user interface	-	-	-	✓	-	-	-	✓
Multi user support	✓	-	-	-	-	-	-	-
Standalone	✓	✓	✓	✓	✓	✓	✓	✓
Web service	✓	-	-	-	-	-	-	-

Compared are several key features of currently available non-commercial exome sequencing analysis pipelines.

n.m. … not mentioned.

a) [14].

b) [15].

c) [16].

d) [17].

e) [18].

f) [50].

g) [19].

## Discussion

SIMPLEX is a novel pipeline for the consistent analysis of exome sequencing data, covering the complete workflow from read filtering and mapping to annotated lists of detected variants. Due to its support for SE and PE data in nucleotide and color space, it is universally applicable to several different platforms and biological problems. Since the installation of all required analysis tools and the targeted analysis itself are still daunting tasks for many researchers, SIMPLEX is provided as a ready to use VirtualBox image and a fully configured Cloud image, which allows users to quickly and comfortably add more computational power. Detected variants are annotated with additional information, including multiple scores which are useful for getting more accurate functional predictions of mutations [31]. The output format was adapted to be easily viewable in standard office software, and a detailed summary report lists several key figures and results of the analysis run.

In principal, it is possible to extend SIMPLEX to support the analysis of whole-genome data by simply changing a few configuration parameters. However, since currently many labs (especially smaller ones) are focusing on exome sequencing data due to the lower data amount and straightforward interpretation of the results, we focused on this data type and included specific steps such as filtering exonic regions, calculation of exome capture specificity, and detection of poorly captured exons.

### Selection of Tools

All external tools that are integrated into SIMPLEX were carefully selected and thoroughly evaluated. Several programs for the read preprocessing steps were tested for their suitability, including TileQC [32], PIQA [33], CANGS [34], and SolexaQA [35]. At the time of development none of them supported both Illumina and ABI SOLiD platforms, and therefore did not qualify for incorporation into the pipeline. As a result, new preprocessing components were developed in-house, which are capable of handling SE and PE reads from Illumina and ABI SOLiD platforms.

Alignment of reads is performed by the Burrows-Wheeler based aligner BWA [21] as it is accurate, fast, open-source and supports gapped as well as quality scored alignment [22]. Furthermore, it is capable of analyzing sequences encoded in nucleotide and color space, handles SE and PE reads, and was selected by the 1000 genome project to map the reads obtained for the *full project* [36]. Other alignment programs such as MAQ [37], SOAP [38], Bowtie [39], and ELAND [40] were ruled out after thorough investigation, as they were either slower than BWA (MAQ, SOAP) or did not support gapped alignment (Bowtie, ELAND). Furthermore, recent studies show that BWA offers superior alignment runtime and memory usage while still providing satisfactory mapping results [41], [42].

Post processing is done either by GATK or by a custom made component which performs several filtering steps. First, it applies mapping and properly-paired filters, followed by PCR duplicate removal and exome filtering. Results of each filtering step are documented in log files and are assembled in a summary report.

GATK was chosen for variant calling, as it supports several NGS platforms and is suitable for both, individual and multi sample, analyses. In addition, the toolkit is shipped with a set of additional SNP analysis tools, including SNP quality evaluation, SNP filtering, and standardized downstream recalibration of variant quality scores. Moreover, it has already been successfully used in large-scale projects like the 1000 Genomes Project [36] and The Cancer Genome Atlas [5]. Amongst other applications tested, SNPseeker [43] is limited to a certain NGS platform, whereas CRISP [44] is only capable of handling pooled sequencing data. Although SAMtools [45] and SOAPsnp [46] are equipped for single sample analysis and multi-platform support, they do not provide functionality to detect the novelty status of SNP calls.

Additional information for detected variants, such as RefSeq names, GO ids, and KEGG ids, are added using the GATK annotator. However, as those annotations depend on existing dbSNP ids, they are not suitable for newly discovered variations. Therefore, SIMPLEX includes Annovar [27] to add additional information for unknown mutations. Annovar was assessed to be superior to other competitors as it comprehends annotations from several databases, provides frequent updates, and is easily extendable. Amongst other evaluated annotation tools, SAAPdb [47] is currently not maintained, *SeattleSeq Annotation* is not available for local installation, and SNPs3D [48] is only available for registered users. The Sequence Variant Analyser [49] does provide several annotations, but is designed as a standalone graphical application and lacks variant scores which are provided by Annovar.

### Comparison with Existing Software

Currently several NGS analysis pipelines are available which differ in their provided feature sets and functionality [14]–[18], [50]. In contrast to several existing pipelines, SIMPLEX is delivered as a fully set up VirtualBox image, which eliminates the arduous installation and setup process of required analysis programs. Furthermore, none of the published pipelines are capable of providing multiple user support and, to the best of our knowledge, none offers the possibility to use current Cloud technologies for data analysis. SIMPLEX covers a complete exome analysis workflow, starting with the analysis of raw sequencing data and ultimately leading to well annotated lists of detected variants. In contrast to other analysis pipelines, detailed summary reports are created at numerous stages which help to quickly evaluate the quality of the sequencing runs and provide an overview of the performed analyses. A comparison overview between pipelines is depicted in Table 5 and Table S3.

### Discussion of Pipeline Evaluation

The performance assessment of the presented pipeline was done with data from the Kabuki study described by [9]. It demonstrated that the application is able to successfully analyze 42 samples in parallel, while still achieving good runtime results (see Table 4). Comparison figures show that more SE reads (63%) than PE reads (54%) could be mapped and more SE reads (23%) than PE reads (16%) were on target.

After the initial analysis, we checked for MLL2 loss-of-function mutations in each individual which could be observed in 8 out of 10 subjects (see Table S4). This number is comparable to the results of the Kabuki study which identified mutations in MLL2 in 9 out of 10 individuals. Furthermore, additional standard Autoannovar analysis labeled MLL2 as a possible candidate mutation in 5 out of 10 individuals (see Tables S5 and S6).

### Conclusion

We have developed and validated SIMPLEX, a highly configurable pipeline for the analysis of NGS exome data, covering the complete workflow from sequence alignment to SNP/DIP identification and variant annotation. Due to the pipeline’s flexible design SIMPLEX is supporting SE and PE data as well as input from various sequencing platforms. The pipeline is optimized for HPC infrastructures and can be used in the Amazon EC2 Cloud to speed up the analysis process. Complex methods and commands are abstracted from investigators to facilitate the use of NGS technologies in laboratories even without a specialized bioinformatics staff. SIMPLEX outputs highly readable reports including summary documents that list key figures such as *exome coverage*, *filtering results*, or *exome capture specificity*. All detected variants are annotated with additional information allowing researchers to easily and quickly discriminate silent mutations from variants that are potentially causing diseases. Furthermore, result files can be used in downstream analyses to additionally identify driver mutations using tools such as Auto-Annovar or VAAST [51]. SIMPLEX combines proven analysis tools with a set of newly developed methods, which are all optimized toward the selection of statistically and functionally significant genetic events. Moreover, the pipeline can be smoothly extended to include additional methods, such as Varscan [52]. In addition, it is generally possible to use SIMPLEX with different organisms and we have outlined all needed steps to include additional species in the user manual. The complete application is continuously tested and is distributed in ready-to-use virtual images that can be easily deployed in the Cloud.

Due to the modular design of the pipeline, it is possible to integrate any command line tool by simply extending a XML file and creating a few Java wrapper classes (for detailed information see the user manual). The whole pipeline code is open-source and can be extended to one’s needs. We hope to encourage the community to create extensions and submit them to the productive development branch. Regarding the long-term use and sustainability of the pipeline, the application is under heavy use in clinical research environment, which guarantees active development and improvements.

In conclusion, SIMPLEX is a tool designed to be readily used for life science researchers to quickly obtain biological insight into genetic events investigated by exome sequencing. The presented system was successfully applied in a recent study to elucidate the cause of the rare genetic disease epileptic encephalopathy and amelogenesis imperfecta (Kohlschütter-Tönz syndrome) [53].

## Supporting Information

Table S1 **SIMPLEX parameters and their description.**
(PDF)

Table S2 **Kabuki syndrome study summary.**
(XLS)

Table S3 **Comparison of SIMPLEX, Atlas, and Treat pipelines.**
(PDF)

Table S4 **Kabuki syndrome study - SNV statistics grouped by individuals.**
(PDF)

Table S5 **Kabuki syndrome study - unique occurrences of MLL2.**
(PDF)

Table S6 **Kabuki syndrome study - summary of autoannovar results.**
(PDF)

Supplementary Material S1 **SIMPLEX user manual.**
(PDF)

Supplementary Material S2 **QA report after raw sequence preprocessing for sample SRR063831.**
(PDF)
