# Supplementary material for: SIMPLEX: Cloud-Enabled Pipeline for the Comprehensive Analysis of Exome Sequencing Data
Source: PLoS One. 2012 Aug 1;7(8):e41948. doi: 10.1371/journal.pone.0041948 (PMC3411592; doi:10.1371/journal.pone.0041948)
Supplement: Table S1 — SIMPLEX parameters and their description. (PDF) [file pone.0041948.s001.pdf]

Supplementary Table 1: SIMPLEX parameter description

Required pipeline parameters

Short option	Long option	Value	Description	Default value
-c	--command	exomeSE | exomePE	defines if singe read or paired end data is analyzed	-
-od	--outputdirectory	text	path to output directory where pipeline results are stored	-
-genP	--genome-prefix	hg18 | hg18.color | hg19 | hg19.color	prefix specifying the reference genome	-
-I	--input	text	comma separated list of fastq files to analyze. If paired end data is given, the file names must contain _R1 or _R2 (for first reads in pair/ second reads in pair) before the format suffix (.fq[.gz] or .fastq[.gz])	-
-sfeb	--sfeb	text	path to bed file specifying the exome	-
-dsP	--dsP	double	percentage to distinguish between homo- and heterozygous DIPs	-
-k	--clusterpropsfile	text	configure access to cluster: path to the clusterproperties file	-

Optional pipeline parameters

Short option	Long option	Type	Description	Default value
-CS	--colorspace	switch	if defined, input files are assumed to be color space csfasta files	-
-Q	--qualfiles	text	list of the quality files in case colorspace csfasta files are used as input. Same rules for file separation apply as for the input files	-
-sfecs	--sf-exon-cs	switch	strand aware exome filtering	-
-gaR	--ga-region	text	defines the region of interest in form of <chrom>:<start>-<end>	-
-ac	--autocleanup-disabled	switch	leaves all files on the cluster for later cleanup	-

FASTQ conversion parameters

Short option	Long option	Type	Description	Default value
-fqc	--fq-convert	switch	enables fastq conversion	-
-cf	--convert-from	illumina | solexa	FASTQ input file format	illumina

Quality statistics parameters

Short option	Long option	Type	Description	Default value
-d	--detailed-results	switch	if given, intermediate results (like parsing results) are fetched	-
-ml	--max-read-length	integer	length of the longest sequence read (needed for parsing)	512
-qo	--qv-offset	integer	FASTQ ASCII encoding offset	33
-qr	--qv-range	integer	max FASTQ Phred quality value	94
-r	--resolution	integer	for the quality report generion, eps figures are converted into pngs (due to file sizes). This parameter defines the png's resolution in dpi	500
-sc	--statistics-command	raw_report | filter_report	comma separated list that defines which fastqstatistics should be calculated	-

Supplementary Table 1: SIMPLEX parameter description

FASTQ read trimmer parameters				
**Short option**	**Long option**	**Type**	**Description**	**Default value**
-ftl	--ft-len	integer	read length to be trimmed to	-
-ftlfp	--ft-len-five-prime	integer	number of bp to be trimmed at 5' position	0
-ftltp	--ft-len-three-prime	integer	number of bp to be trimmed at 3' position	0
-ftq	--ft-qual	integer	numeric quality value to be trimmed in the quality string.	-
-ftS	--ft-seq	char	character to be trimmed in the sequence string.	-

FASTQ read filter parameters				
**Short option**	**Long option**	**Type**	**Description**	**Default value**
-ffqf	--ff-qf	double/integer	maximum amount of allowed values of the specified quality value in the read. *Double* between 0 and 1 are treated as percent, otherwise *integer* (=total amount of Ns) is expected.	-
-ffqv	--ff-qv	integer	numerical quality value to be filtered	-
-M	--maxl	integer	maximal length of a sequence	-
-m	--minl	integer	minimal length of a sequence	-
-N	--nmax	double/integer	maximum amount of allowed Ns in a sequence. *Double* between 0 and 1 are treated as percent, otherwise *integer* (=total amount of Ns) is expected.	-

Bwa aln parameters				
**Short option**	**Long option**	**Type**	**Description**	**Default value**
-bwaad	--bwaad	integer	disallow a long deletion within *integer* bp towards the 3'-end	16
-bwaae	--bwaae	integer	maximum number of gap extensions, -1 for k-difference mode (disallowing long gaps)	-1
-bwaaE	--bwaaE	integer	gap extension penalty	4
-bwaai	--bwaai	integer	disallow an indel within *integer* bp towards the ends	5
-bwaak	--bwaak	integer	maximum edit distance in the seed	2
-bwaal	--bwaal	integer	take the first *integer* subsequence as seed. If *integer* is larger than the query sequence, seeding will be disabled. For long reads, this option is typically ranged from 25 to 32 for '*bwaak*-2'.	inf
-bwaaM	--bwaaM	integer	mismatch penalty. BWA will not search for suboptimal hits with a score lower than (bestScore-*integer*).	3
-bwaan	--bwaan	double/integer	maximum edit distance if the value is *integer*, or the fraction of missing alignments given 2% uniform base error rate if *double*. In the latter case, the maximum edit distance is automatically chosen for different read lengths.	0.04
-bwaaN	--bwaaN	switch	disable interactive search. All hits with no more than *bwaan* differences will be found. This mode is much slower than the default.	-
-bwaao	--bwaao	integer	maximum number of gap opens	1

Supplementary Table 1: SIMPLEX parameter description

-bwaaO	--bwaaO	integer	gap open penalty	11
-bwaaq	--bwaaq	integer	parameter for read trimming. BWA trims a read down to argmax_x{\sum{i0x+1}^l(*integer*-q_i)} if q_l<*integer* where l is the original read length.	0
-bwaar	--bwaar	integer	proceed with suboptimal alignments if there are no more than *integer* equally best hits. This option only affects paired-end mapping. Increasing this threshold helps to improve the pairing accuracy at the cost of speed, especially for short reads (~32bp).	30

Bwa samse parameters				
**Short option**	**Long option**	**Type**	**Description**	**Default value**
-bwassn	--bwassn	integer	maximum number of alignments to output in the XA tag for reads paired properly. If a read has more than *integer* hits, the XA tag will not be written.	3

Bwa sampe parameters				
**Short option**	**Long option**	**Type**	**Description**	**Default value**
-bwaspA	--bwaspA	switch	disable insert size estimate (force *-bwasps*)	-
-bwaspa	--bwaspA	integer	maximum insert size	500
-bwaspc	--bwaspc	double	prior of chimeric rate	0.00001
-bwaspn	--bwaspn	integer	maximum number of alignments to output in the XA tag for reads paired properly. If a read has more than *integer* hits, the XA tag will not be written.	3
-bwaspN	--bwaspn	integer	maximum number of alignments to output in the XA tag for disconcordant read pairs (excluding singletons). If a read has more than *integer* hits, the XA tag will not be written.	10
-bwaspo	--bwaspo	integer	maximum occurrences for one end	100000
-bwaspp	--bwaspp	switch	preload index into memory (for base-space reads only)	-
-bwasps	--bwasps	switch	disable Smith-Waterman for the unmapped mate	-

Local realignment parameters				
**Short option**	**Long option**	**Type**	**Description**	**Default value**
-mlret	--mlr-entropy-thr	double	percentage of mismatching base quality scores at a position to be considered having high entropy.	0.15
-mlrid	--mlr-indels	chr:start-end	list of already known indels	dbSNP indels
-mlrko	--mlr-knowns-only	switch	don't run Smith-Waterman to generate alternate consenses; use only known indels provided as RODs for constructing the alternate references.	-

-mlrlod	--mlr-lod	double	LOD threshold above which the realigner will proceed to realign. This term is equivalent to significance - e.g. is the improvement significant enough to merit realignment? Note that this number should be adjusted based on your particular data set. For low coverage and /or when looking for indels with low allele frequency, this number should be smaller.	5.0
-mlrmc	--mlr-max-cons	integer	maximum alternate consensuses to try (necessary to improve performance in deep coverage). If you need to find the optimal solution regardless of running time, use a higher number.	30
-mlrmis	--mlr-max-interval-size	integer	max size in base pairs of intervals that will be passed to the realigner. Because the realignment algorithm is N^2, allowing too large an interval might take too long to completely realign.	500
-mlrmlr	--mlr-min-loc-reads	integer	minimum coverage at a locus for the entropy calculation to be enabled.	4
-mlrmmf	--mlr-mismatch-fract	double	fraction o f total sum of base qualities at a position that need to mismatch for the position to be considered to have high entropy. Note that this fraction should be adjusted based on your particular data set. For deep coverage and/or when looking for indels with low allele frequency, this number should be smaller than the default value.	0.15
-mlrmrc	--mlr-max-reads-cons	integer > 0	maximum number of reads (chosen randomly) used for finding the potential alternate consensuses (necessary to improve performance in deep coverage). If you need to find the optimal solution regardless of running time, use a higher number.	120
-mlrws	--mlr-window-size	integer > 0	any two SNP calls and/or high entropy positions are considered clustered when they occur no more than N base pairs apart.	10

**Mark duplicates parameters**				
**Short option**	**Long option**	**Type**	**Description**	**Default value**
-mdd	--md-pixel-dist	integer > 0	the maximum offset between two duplicate clusters in order to consider them optical duplicates. This should usually be set to some fairly small number (e.g. 5-10 pixels) unless using later versions of the Illumina pipeline that multiply pixel values by 10, in which case 50-100 is more normal.	100
-mdr	--md-rn-regex	text	regular expression that can be used to parse read names in the incoming SAM file. Read names are parsed to extract three variables: tile/region, x coordinate and y coordinate. These values are used to estimate the rate of optical duplication in order to give a more accurate estimated library size. The regular expression should contain three capture groups for the three variables, in order.	[a-zA-Z0-9]+:[0-9]:([0-9]+):([0-9]+):([0-9]+).*
-mds	--md-max-seqs	integer > 0	the maximum number of sequences allowed in SAM file. If this value is exceeded, program won't spill to disk (used to avoid situation where there are not enough file handles).	50000

Supplementary Table 1: SIMPLEX parameter description

-nd	--no-dup	switch	if given, duplicate removal is not preformed, available for SE mode only.	-
-pmr	--picard-max-ram	integer	this specifies the number of records stored in RAM before spilling to disk. Increasing this number reduces the number of file handles needed and increases the amount of RAM needed.	500000

Exon filter parameters				
**Short option**	**Long option**	**Type**	**Description**	**Default value**
-sfecs	--sf-exon-cs	switch	if set - strand has to match (+/-) strand to pass the filter.	-
-skip	--skip-trace	switch	skip writing intermediate failed sequence outputs.	-

Alignment summary parameters				
**Short option**	**Long option**	**Type**	**Description**	**Default value**
-casmmi	--casm-max-insert	integer	paired end reads above this insert size will be considered chimeric along with inter-chromosomal pairs.	100000
-pmr	--picard-max-ram	integer	this specifies the number of records stored in RAM before spilling to disk. Increasing this number reduces the number of file handles needed and increases the amount of RAM needed.	500000

DIP genotyping parameters				
**Short option**	**Long option**	**Type**	**Description**	**Default value**
-dipcmc	--dipc-min-cov	integer > 0	indel calls will be made only at sites with coverage of minCoverage or more reads.	6
-dipcmcf	--dipc-min-cons-frac	double Є [0;1]	indel call is made only if fraction of consensus indel observations at a site with respect to all indel observations at the site exceeds this threshold.	0.7
-dipcmf	--dipc-min-frac	double Є [0;1]	minimum fraction of reads with consensus indel at a site, out of all reads covering the site, required for making a call (fraction of non-consensus indels at the site is not considered here, see *-dipcmcf*).	0.3
-dipcmic	--dipc-min-indel-count	integer ≥ 0	minimum count of reads supporting consensus indel required for making the call. This filter supercedes *dipcmf*, i.e. indels with acceptable *dipcmf* at low coverage (*dipcmic* not met) will not pass.	0
-dipcmr	--dipc-max-reads	integer	maximum number of reads to cache in the window; if number of reads exceeds this number, the window will be skipped and no calls will be made from it.	-

Supplementary Table 1: SIMPLEX parameter description

-dipcws	--dipc-window-size	integer > 0	in order to be able to 1) count in all indel- and reference-supporting reads and to collect alignment statistics (mismatches, base quals etc) for each putative event 2) resolve nearby putative events (spanned by a read) and (re-)compute all stats for each of them, the genotyper caches the reads inside a sliding window. The window must be definitely larger than the longest span of a read on the reference (note: alignments with long deletions will have large span (read length + deletion length)), 2-3 times the read length is usually more than enough.	200
-dipcmcavmm	--dipc-max-cons-av-mm	double ≥ 0	max. average number of mismatches per (consensus) indel-containing read. If the number is greater than this threshold, indel will be discarded/marked.	3.0
-dipcmcavq	--dipc-min-cons-av-qual	double ≥ 0	min. average base quality in all indel supporting reads in the nqs window around the indel. If the average base quality is less than this threshold, the indel will be discarded/ marked.	0.0
-dipcmcnqsmm	--dipc-max-cons-nqs-mm	double ≥ 0	max. average mismatch rate in NQS window around the indel, across all indel-containing read. If the number is greater than this threshold, indel will be discarded/marked.	0.5
-dipcmravmm	--dipc-max-ref-av-mm	double ≥ 0	max. average number of mismatches per reference-matching read. If the number is greater than this threshold, indel will be discarded/marked.	100000
-dipcmrnq	--dipc-min-ref-nq	double ≥ 0	min. average base quality in all reference supporting reads in the nqs window around the indel. If the average base quality is less than this threshold, the indel will be discarded/ marked	0.0
-pmr	--picard-max-ram	integer	this specifies the number of records stored in RAM before spilling to disk. Increasing this number reduces the number of file handles needed and increases the amount of RAM needed.	500000

**SNP genotyping parameters**				
**Short option**	**Long option**	**Type**	**Description**	**Default value**
-snpcab	--snpc-all-bases	switch	instructs the genotyper to emit calls at all bases with coverage, regardless of the confidence or genotype at the locus.	-
-snpcbm	--snpc-base-model	ONE_STATE | THREE_STATE | EMPIRICAL	base substitution model to employ	EMPIRICAL
-snpccbq	--snpc-cap-base-qual	switch	cap the base quality of any given base by its read's mapping quality.	-
-snpcd	--snpc-del	double	maximum fraction of reads with deletions spanning this locus for it to be callable (to disable, set to < 0 or > 1).	0.05
-snpcg	--snpc-genotype	switch	enables genotyping mode, whereby the confidence in the genotype itself is used for the confidence threshold test rather than the confidence in a non-reference genotype. Should the output be confident genotypes (i.e. including ref calls) or just the variants?	-

Supplementary Table 1: SIMPLEX parameter description

-snpcgm	-snpcgm	GM_JOINT_ESTIMATE | GM_DINDEL	genotype calculation model to employ.	GM_JOINT_ESTIMATE
-snpch	--snpc-het	double	value used to compute prior likelihoods for any locus.	0.001
-snpcmbq	--snpc-min-base-qual	integer ≥ 0	minimum base quality required to consider a base for calling.	10
-snpcmmmiw	--snpc-max-mm-in-window	integer ≥ 0	maximum number of mismatches within a 40 bp window (20bp on either side) around the target position for a read to be used for calling.	3
-snpcmmq	--snpc-min-mq	integer ≥ 0	minimum read mapping quality required to consider a read for calling.	10
-snpcmr	--snpc-max-reads	integer ≥ 0	specifies the maximum coverage at a locus. This is used to skip loci that have too much coverage.	-
-snpcns	--snpc-no-SLOD	switch	instructs the genotyper not to calculate the SLOD.	-
-snpcscc	--snpc-std-call-conf	integer ≥ 0	the minimum phred-scaled confidence threshold at which variants not at 'trigger' track sites should be called.	30
-snpcsec	--snpc-std-emit-conf	integer ≥ 0	the minimum phred-scaled confidence threshold at which variants not at 'trigger' track sites should be emitted (and marked as filtered if less than the calling threshold).	10
-snpctcc	--snpc-trig-call-conf	integer ≥ 0	the minimum phred-scaled confidence threshold at which variants at 'trigger' track sites should be called.	30
-snpctec	--snpc-trig-emit-conf	integer ≥ 0	the minimum phred-scaled confidence threshold at which variants at 'trigger' track sites should be emitted (and marked as filtered if less than the calling threshold).	10
-snpfc	--snpf-cluster	integer ≥ 0	number of SNPs which make up a cluster	3
-snpfcs	--snpf-cluster-size	integer ≥ 0	window size (in bases) in which to evaluate clustered SNPs (to disable the clustered SNP filter, set this value to less than 1	0
-snpfmw	--snpf-mask-window	integer ≥ 0	number of bases to extend an indel interval on both sides	10

**variant quality score recalibration parameters**				
**Short option**	**Long option**	**Type**	**Description**	**Default value**
-rvqavcfdr	--rvq-avc-fdr	double	ApplyVariantCuts - fdr_filter_level: The FDR level at which to start filtering.	10
-rvqb	--rvq-vr-bO	double	VariantRecalibrator - backOff: The Gaussian back off factor, used to prevent overfitting by enlarging out the Gaussians.	1.3
-rvqd	--rvq-gvc-d	double	GenerateVariantClusters - dirichlet: the dirichlet parameter in variational Bayes algoirthm.	1000.0
-rvqD	--rvq-gvc-wD	double	GenerateVariantClusters - weightDBSNP: the weight for dbSNP variants during clustering.	0.0
-rvqdV	--rvq-vr-dv	integer	VariantRecalibrator - dV: The desired number of variants to keep in a theoretically filtered set.	0
-rvqfdr	--rvq-vr-fdr	comma separated doubles	VariantRecalibrator - FDRtranche: comma separed list of levels of novel false discovery rate (FDR, implied by ti/tv) at which to slice the data. (in percent, that is 1.0 for 1 percent).	-

Supplementary Table 1: SIMPLEX parameter description

-rvqfI	--rvq-gvc-fI	switch	GenerateVariantClusters - forceIndependent: force off-diagonal entries in the covariance matrix to be zero.	-
-rvqg	--rvq-gvc-mG	integer	GenerateVariantClusters - mG: the maximum number of Gaussians to try during Bayesian clustering	4
-rvqH	--rvq-gvc-wH	double	GenerateVariantClusters - weightHapMap: the weight for HapMap variants during clustering.	1.0
-rvqi	--rvq-gvc-mI	integer	GenerateVariantClusters - mI: the maximum number of iterations to be performed when clustering. Clustering will normally end when convergence is detected.	200
-rvqk	--rvq-gvc-u1kg	switch	GenerateVariantClusters: use 1000 genomes project data to generate variant clusters.	-
-rvqK	--rvq-gvc-wK	double	GenerateVariantClusters - weight1KG: The weight for 1000 Genomes Project variants during clustering.	1.0
-rvqn	--rvq-gvc-wN	double	GenerateVariantClusters - weightNovel: the weight for novel variants during clustering.	0.0
-rvqpD	--rvq-vr-pD	double	VariantRecalibrator - priorDBSNP: A prior on the quality of dbSNP variants, a phred scaled probability of being true.	10.0
-rvqpH	--rvq-vr-pH	double	VariantRecalibrator - priorHapMap: A prior on the quality of HapMap variants, a phred scaled probability of being true.	15.0
-rvqpK	--rvq-vr-pK	double	Genomes Project variants, a phred scaled probability of being true. Currently not supported since 1000 Genomes Project data is not on cluster yet.	12.0
-rvqpN	--rvq-vr-pN	double	VariantRecalibrator - priorNovel: A prior on the quality of novel variants, a phred scaled probability of being true.	2.0
-rvqQ	--rvq-gvc-q	integer	GenerateVariantClusters - qual: if a known variant has raw QUAL value less than -qual then don't use it for clustering	-
-rvqQ	--rvq-vr-qstep	double	VariantRecalibrator - qStep: Resolution in QUAL units for optimization and tranche calculations.	0.1
-rvqs	--rvq-gvc-s	double	GenerateVariantClusters - shrinkage: the shrinkage parameter in variational Bayes algorithm.	0.0001
-rvqS	--rvq-vr-qscale	double	VariantRecalibrator - qScale: Multiply all final quality scores by this value. Needed to normalize the quality scores.	100.0
-rvqsfp	--rvq-vr-sfp	double	VariantRecalibrator - singleton_fp_rate: Prior expectation that a singleton call would be a FP.	0.5
-rvqt	--rvq-gvc-std	double	GenerateVariantClusters - std: if a variant has annotations more than -std standard deviations away from mean then don't use it for clustering.	4.5
-rvqT	--rvq-vr-titv	double	VariantRecalibrator - titv: The expected novel Ti/Tv ratio to use when calculating FDR tranches and for display on optimization curve output figures. (~2.07 for whole genome experiments; 3.0 for whole exome experiments)	3.0

Supplementary Table 1: SIMPLEX parameter description

Supplementary Table 1: SIMPLEX parameter description

Supplementary Table 1: SIMPLEX parameter description

Supplementary Table 1: SIMPLEX parameter description

Supplementary Table 1: SIMPLEX parameter description

Supplementary Table 1: SIMPLEX parameter description

Supplementary Table 1: SIMPLEX parameter description

Supplementary Table 1: SIMPLEX parameter description
