# Supplementary material for: SIMPLEX: Cloud-Enabled Pipeline for the Comprehensive Analysis of Exome Sequencing Data
Source: PLoS One. 2012 Aug 1;7(8):e41948. doi: 10.1371/journal.pone.0041948 (PMC3411592; doi:10.1371/journal.pone.0041948)
Supplement: Table S3 — Comparison of SIMPLEX, Atlas, and Treat pipelines. (PDF) [file pone.0041948.s003.pdf]

Supplementary Table 3: Comparison Simplex-Atlas2-Treat

	**SIMPLEX**	**Atlas2 (filtered)**	**Intersection Simplex-Atlas2**	**Treat**
SNP – SE	2,588	7,025	1,480	- *
SNP – PE	3,539	6,779	1,516	56,073 **
DIP – SE	173	18,735	145	- *
DIP – PE	370	55,390	333	3,534 **
**Runtime(min)**				
SNP – SE	5	37	-	- ***
SNP – PE	6	56	-	- ***
DIP – SE	3	28	-	- ***
DIP – PE	5	44	-	- ***

* SE data was not tested as TREAT threw an error.

** Treat vcf results are called “*raw.vcf” and do not contain any values in the filter column. Therefore, we suggest that the resulting files respresent raw SNPs.

*** Runtime for Treat could not be established since the pipeline resulted in an error.
