# Supplementary material for: SIMPLEX: Cloud-Enabled Pipeline for the Comprehensive Analysis of Exome Sequencing Data
Source: PLoS One. 2012 Aug 1;7(8):e41948. doi: 10.1371/journal.pone.0041948 (PMC3411592; doi:10.1371/journal.pone.0041948)
Supplement: Table S4 — Kabuki syndrome study - SNV statistics grouped by individuals. (PDF) [file pone.0041948.s004.pdf]

Supplementary Table 4: Individual statistics

Individual	Sample	Type	SNPs	DIPs	Transitions (Ti)	Transversions (Tv)	Ti – Tv Ratio	AutoA	MLL2 mut.	MLL 2 Loss-of-function
2441			8414/8414	1051/1050	6462	1952	3.31		6	0
	SRR063756	SE	4790	238	3676	1114	3.30		1	
	SRR063757	SE	4105	197	3147	958	3.28		3	
	SRR063758	SE	7331	573	5645	1686	3.35		4	
	SRR063759	SE	3573	608	2757	816	3.38		6	
2450			13720/13720	1840/1837	10571	3149	3.36		6	0
	SRR063823	SE	3704	892	2855	849	3.36		3	
	SRR063827	SE	5358	871	4071	1287	3.16		4	
	SRR063831	SE	2392	521	1822	570	3.20		1	
	SRR063836	SE	13597	875	10484	3113	3.37		3	
	SRR063842	SE	4027	749	3065	962	3.19		6	
2485			10956/10956	1379/1379	8445	2511	3.36		8	8
	SRR063822	SE	8604	586	6662	1942	3.43		5	
	SRR063825	SE	8070	582	6194	1876	3.30		5	
	SRR063829	SE	7157	818	5490	1667	3.29		5	
	SRR063837	SE	5507	564	4225	1282	3.30		5	
2570			16115/16114	1596/1593	9700	2917	3.33		8	8
	SRR063821	SE	7502	533	5711	1791	3.19		6	
	SRR063828	SE	8687	796	6621	2066	3.20		5	
	SRR063835	SE	8902	736	6839	2063	3.32		7	
	SRR063838	SE	8315	385	6419	1896	3.39		8	
	SRR063841	SE	14904	791	11341	3563	3.18		5	
2817			5764/5763	799/799	4406	1358	3.24	1	11	3
	SRR063834	SE	2588	173	1967	621	3.17		5	
	SRR063839	SE	4199	161	3254	945	3.44		5	
	SRR063843	PE	3539	370	2715	824	3.29	+	11	

	SRR063852	PE	3734	472	2892	842	3.43		7	
2998			15698/15696	924/924	12119	3579	3.39	2	13	3
	SRR063826	SE	7992	242	6233	1759	3.54		6	
	SRR063833	SE	7220	281	5625	1595	3.53		4	
	SRR063847	PE	14847	479	11496	3351	3.43	+	9	
	SRR063848	PE	10190	470	7901	2289	3.45	+	8	
3033			6582/6582	836/835	5006	1576	3.18	3	8	6
	SRR063824	SE	4565	252	3471	1094	3.17	+	2	
	SRR063830	SE	4229	235	3262	967	3.37			
	SRR063845	PE	4818	387	3709	1109	3.34	+	5	
	SRR063849	PE	2981	304	2323	658	3.53	+	7	
3213			10005/10003	694/694	7381	2624	2.81	1	7	2
	SRR063832	SE	5594	242	4297	1297	3.31		2	
	SRR063840	SE	5447	210	4212	1235	3.41	+	2	
	SRR063846	PE	6840	440	5218	1622	3.22		3	
	SRR063850	PE	3260	19	2226	1034	2.15		3	
3566			13754/13754	894/894	10632	3122	3.41		10	4
	SRR063819	SE	6590	291	5036	1554	3.24		4	
	SRR063820	SE	4507	223	3442	1065	3.23		6	
	SRR063844	PE	12649	547	9815	2834	3.46		7	
	SRR063851	PE	5761	246	4501	1260	3.57		5	
3579			15071/15068	1054/1053	11621	3450	3.37	1	10	2
	SRR063943	SE	3489	249	2706	783	3.46		5	
	SRR063944	SE	7750	316	6059	1691	3.58		3	
	SRR063945	PE	13115	544	10156	2959	3.43		8	
	SRR063946	PE	12771	554	9892	2879	3.44	+	7	

(shaded)		… unique per individual
SNPs/DIPs	per individual	… first = union variants where type of mutation is different; second = union variants where type of mutation is not considered
AutoA		… occurrences of MLL2 detected by autoannotator (run with default settings)
