# Supplementary material for: SIMPLEX: Cloud-Enabled Pipeline for the Comprehensive Analysis of Exome Sequencing Data
Source: PLoS One. 2012 Aug 1;7(8):e41948. doi: 10.1371/journal.pone.0041948 (PMC3411592; doi:10.1371/journal.pone.0041948)
Supplement: Table S5 — Kabuki syndrome study - unique occurrences of MLL2. (PDF) [file pone.0041948.s005.pdf]

Supplementary Table 5: Unique occurrences of MLL2

#	Chrom	Start	End	Type	Subst
13	chr12	49416046	49416047	DIP	TC-T
4	chr12	49418435	49418435	SNP	A-C
1	chr12	49418442	49418442	SNP	T-G
3	chr12	49420131	49420131	SNP	A-C
4	chr12	49420554	49420554	SNP	C-T
1	chr12	49420658	49420658	SNP	G-A
1	chr12	49420977	49420977	SNP	A-G
1	chr12	49424393	49424393	SNP	C-G
1	chr12	49424425	49424425	SNP	G-A
2	chr12	49424534	49424534	SNP	G-A
2	chr12	49424703	49424703	SNP	G-A
2	chr12	49424946	49424946	DIP	C-CA
3	chr12	49425575	49425575	SNP	C-T
3	chr12	49425791	49425791	SNP	G-A
22	chr12	49425978	49425978	SNP	T-C
2	chr12	49426404	49426404	SNP	G-A
5	chr12	49426460	49426460	SNP	A-G
8	chr12	49426515	49426515	SNP	C-T
1	chr12	49426592	49426592	SNP	A-G
3	chr12	49426690	49426694	DIP	TGTTG-T
1	chr12	49426896	49426896	SNP	G-C
28	chr12	49427652	49427652	SNP	C-T
4	chr12	49427919	49427919	SNP	T-C
1	chr12	49428440	49428440	SNP	C-A
1	chr12	49431598	49431598	SNP	C-A
4	chr12	49432651	49432651	SNP	G-A
1	chr12	49433040	49433040	SNP	T-G
1	chr12	49433128	49433128	SNP	A-G
1	chr12	49433760	49433760	SNP	C-G
21	chr12	49434074	49434074	SNP	C-A
1	chr12	49434393	49434393	SNP	G-A
1	chr12	49434509	49434509	SNP	C-T
1	chr12	49434621	49434621	SNP	G-C
1	chr12	49435157	49435157	SNP	G-T
3	chr12	49435258	49435258	SNP	G-A
3	chr12	49435971	49435971	SNP	G-A
1	chr12	49436106	49436106	SNP	C-A
1	chr12	49438041	49438041	SNP	C-A
3	chr12	49442561	49442563	DIP	CAG-C
4	chr12	49443785	49443785	DIP	G-GT
27	chr12	49444545	49444545	SNP	G-A
4	chr12	49445028	49445028	SNP	G-A
2	chr12	49445069	49445069	SNP	A-G
2	chr12	49445092	49445092	SNP	C-G
1	chr12	49445104	49445104	SNP	A-G
1	chr12	49445148	49445148	SNP	T-C
1	chr12	49445181	49445181	SNP	G-C
1	chr12	49447302	49447302	SNP	C-T
3	chr12	49448463	49448463	SNP	C-T
