# Supplementary material for: SIMPLEX: Cloud-Enabled Pipeline for the Comprehensive Analysis of Exome Sequencing Data
Source: PLoS One. 2012 Aug 1;7(8):e41948. doi: 10.1371/journal.pone.0041948 (PMC3411592; doi:10.1371/journal.pone.0041948)
Supplement: Table S6 — Kabuki syndrome study - summary of autoannovar results. (PDF) [file pone.0041948.s006.pdf]

Supplementary Table 6: Genes detected by annovar

Occurrences	Gene
28	CDC27
17	RBMX
13	PABPC1
12	ZNF626
12	MN1
11	KRTAP5
10	DSPP
9	KRT10
9	HNRNPUL1
8	SPTBN4
8	HMCN1
8	BMP2K
7	ZFYVE9
7	ZFHX3
7	SEPT6
7	MLL2
7	DOCK11
7	ATXN2
6	VPS13B
6	TDG
6	SYNE2
6	SFPQ
6	PLEC
6	NEB
6	MCF2
6	IGFBPL1
6	FAM155A
6	DYNC2H1
6	CDK13
6	BRWD3
6	BIRC6
5	ZNF493
5	ZFC3H1
5	UBR4
5	UBR1
5	SRCAP
5	SON
5	SMARCA2
5	S100A1
5	PKHD1L1
5	PKD1L2
5	NRG2
5	NOX1
5	MEGF6
5	MAGEC1
5	LATS1
5	HUWE1
5	HERC1
5	GPR98
5	FAT1

5	DST
5	DMD
5	CSMD3
5	CASZ1
5	CAMKV
5	ATG2A
5	AMELY
4	ZRANB3
4	ZNF281
4	WDR13
4	VPS13A
4	UTRN
4	USP34
4	USH2A
4	URB1
4	TNRC18
4	TFDP3
4	SORCS3
4	SIRPB1
4	SETD2
4	RXRA
4	ROS1
4	RBBP6
4	PCLO
4	ODZ3
4	MYOF
4	MLL
4	LYST
4	LRP2
4	LRBA
4	KNDC1
4	KIF21A
4	FAT4
4	DOCK6
4	DNAH8
4	DNAH7
4	DNAH3
4	DNAH2
4	CTDSP1
4	COL24A1
4	COL12A1
4	CHD9
4	C8orf38
4	C16orf11
4	BOD1L
4	ATG2B
4	APC2
4	AKAP9
3	ZNF85
3	ZNF828
3	ZNF74
3	ZNF638
3	ZNF430

3	ZNF192
3	ZBTB4
3	YTHDC2
3	XIRP2
3	WDR7
3	WDR43
3	WDR19
3	VPS8
3	VPS54
3	VPS13D
3	VPS13C
3	USP9X
3	USP5
3	USP32
3	UNC79
3	UNC13D
3	ULK1
3	UGGT2
3	TTC3
3	TTC17
3	TRRAP
3	TRPM2
3	TPT1
3	TPR
3	TNIK
3	TDRD6
3	SYNE1
3	SRBD1
3	SPTA1
3	SMG1
3	SMCHD1
3	SMC2
3	SLC7A7
3	SLC3A1
3	SLC30A5
3	SI
3	SETD5
3	SEC22B
3	SDK2
3	SCN1A
3	SCAF4
3	SAFB2
3	SACS
3	RYR3
3	RREB1
3	ROBO2
3	RLF
3	RELT
3	RELN
3	RCBTB1
3	RBM33
3	RBM25
3	RASGRF2

3	QSER1
3	PWWP2A
3	PSMD7
3	PRKAA1
3	PRIC285
3	POLR3D
3	POLR2A
3	POLE
3	PNPLA6
3	PLEKHH2
3	PLCH1
3	PKHD1
3	PIK3C2A
3	PHIP
3	PER1
3	PDS5B
3	PCNX
3	PCDHAC1
3	PCDH7
3	PCDH15
3	NUFIP2
3	NEURL4
3	NCOA6
3	NBEAL1
3	NBAS
3	MYT1L
3	MYO16
3	MYH10
3	MTMR10
3	MRPS17
3	MICAL3
3	MED9
3	MED12L
3	MDN1
3	MCCC2
3	MAP3K4
3	MAP1S
3	MACF1
3	LRPPRC
3	LRP4
3	LRP1B
3	LRFN4
3	LOXL4
3	LHFPL1
3	LARS2
3	KIF1B
3	KIF14
3	KIAA2026
3	KIAA1429
3	KIAA1109
3	KIAA0564
3	JMJD1C
3	IFT140

3	HOOK2
3	HNRNPU
3	HECTD1
3	HEATR5B
3	GUCY2C
3	GPR112
3	GOLGB1
3	GIPC3
3	GCA
3	GBX1
3	GAPVD1
3	GAK
3	FRYL
3	FREM2
3	FBN1
3	FAT3
3	FAM83H
3	FAM40A
3	FAM189B
3	FAM120C
3	EVPL
3	EP400
3	EIF5B
3	EIF4G3
3	EEA1
3	EDC3
3	DSEL
3	DOPEY2
3	DNMBP
3	DNAH5
3	DMXL2
3	DMXL1
3	DMTF1
3	DDX46
3	DDX26B
3	CTAGE5
3	COL7A1
3	COL5A2
3	COL4A4
3	COL4A3
3	COL3A1
3	COL16A1
3	COL14A1
3	COL11A2
3	COL11A1
3	CNTRL
3	CNOT1
3	CLTC
3	CHD6
3	CHD5
3	CENPE
3	CCNB3
3	CARD10

3	CADPS2
3	C7orf51
3	C6orf170
3	BRWD1
3	ATRX
3	ATP8B1
3	ATP7A
3	ATF7IP
3	ASTN2
3	ARMC3
3	ARIH1
3	AR
3	APC
3	APBB1IP
3	APBB1
3	ANK2
3	ANK1
3	AHNAK
3	AGTPBP1
3	ADCK5
3	ADAMTS9
3	ACTRT2
3	ABCA12
2	ZSWIM6
2	ZSWIM4
2	ZNFX1
2	ZNF91
2	ZNF771
2	ZNF732
2	ZNF695
2	ZNF608
2	ZNF573
2	ZNF566
2	ZNF536
2	ZNF507
2	ZNF486
2	ZNF469
2	ZNF468
2	ZNF441
2	ZNF429
2	ZNF420
2	ZNF283
2	ZNF267
2	ZNF236
2	ZMYM2
2	ZMIZ2
2	ZHX3
2	ZCCHC7
2	ZCCHC11
2	ZC3H7A
2	ZC3H11A
2	YTHDC1
2	XYLT1

2	XRN1
2	XPO5
2	XPO1
2	WDR90
2	WDR72
2	WDR47
2	WDR33
2	WDHD1
2	WDFY3
2	WASL
2	VWA5B1
2	VSX1
2	VPS37D
2	VPS37A
2	VIM
2	UTP3
2	USP54
2	USP45
2	USP40
2	USP4
2	USP38
2	USP36
2	UNC80
2	UNC5B
2	UHRF1BP1
2	UGP2
2	UGGT1
2	UBE2O
2	TULP4
2	TTYH3
2	TTPA
2	TTLL7
2	TTLL11
2	TTBK2
2	TTBK1
2	TSSK3
2	TSPYL2
2	TRPC3
2	TRIOBP
2	TRIO
2	TRIM47
2	TRIM36
2	TRIM2
2	TRAPPC8
2	TRAK2
2	TPP2
2	TPMT
2	TPH2
2	TP53BP1
2	TOP2B
2	TNFAIP3
2	TNC
2	TMPRSS2

2 TMEM48
2 TMEM33
2 TM9SF2
2 TKT
2 TJP1
2 TIGD1
2 TG
2 TEX10
2 TERT
2 TBX6
2 TBX5
2 TBC1D16
2 TAPT1
2 TAOK2
2 TANC2
2 TAF5
2 TAF2
2 SZT2
2 SYNRG
2 SYAP1
2 SVEP1
2 SULT1C4
2 STXBP5
2 STIL
2 STAT3
2 SSX2IP
2 SSH1
2 SRRM4
2 SRR
2 SRP68
2 SPTBN2
2 SPTAN1
2 SPPL2A
2 SPG11
2 SPEG
2 SPAST
2 SPAG9
2 SPAG17
2 SOX3
2 SORL1
2 SNX7
2 SNX13
2 SMURF2
2 SMG9
2 SMEK1
2 SMC5
2 SMARCAL1
2 SMARCA4
2 SLK
2 SLC9A1
2 SLC8A3
2 SLC5A8
2 SLC4A7

2	SLC4A3
2	SLC4A1AP
2	SLC39A6
2	SLC28A2
2	SLC25A35
2	SLC1A4
2	SIPA1L2
2	SIPA1L1
2	SIK2
2	SHROOM4
2	SHCBP1L
2	SHANK1
2	SH3BP5
2	SGOL2
2	SF3B1
2	SETMAR
2	SETD1A
2	SEC31B
2	SEC23IP
2	SDCCAG8
2	SCRIB
2	SCN9A
2	SAMD9
2	RYR1
2	RTTN
2	RTN4RL2
2	RTN4
2	RPP40
2	RPL19
2	RP1L1
2	RNMT
2	RNF219
2	RNF20
2	RMND5A
2	RIMS2
2	RICTOR
2	RGS9
2	RGS22
2	RGS12
2	RFTN2
2	REV3L
2	RBM46
2	RBM26
2	RB1CC1
2	RAPGEF4
2	RANGAP1
2	RALGAPA2
2	RABGAP1
2	PVRL4
2	PUS7
2	PTPN21
2	PTK2B
2	PSTPIP1

2	PSME4
2	PRRC2B
2	PRRC2A
2	PRR12
2	PRPSAP1
2	PRPF40B
2	PRPF38A
2	PRICKLE3
2	PREX2
2	PPL
2	PPFIA1
2	POU4F2
2	POLR2B
2	POLQ
2	POLD1
2	POLA1
2	PNN
2	PMS1
2	PLXNA4
2	PLRG1
2	PLCB4
2	PIK3R2
2	PIK3CD
2	PICALM
2	PIBF1
2	PHKA1
2	PHC1
2	PHACTR2
2	PFKFB1
2	PFAS
2	PDZRN3
2	PDF
2	PDE4A
2	PCM1
2	PCF11
2	PCDH12
2	PBX1
2	PAXIP1
2	PALM3
2	PACS1
2	OTUD4
2	OSBPL8
2	ODZ4
2	ODZ1
2	NXF1
2	NUP98
2	NUP210L
2	NUP188
2	NSUN6
2	NSD1
2	NRXN2
2	NRAP
2	NPR3

2	NPHS1
2	NOTCH3
2	NOTCH1
2	NOL10
2	NMT2
2	NKX3
2	NIPBL
2	NIN
2	NFS1
2	NFIB
2	NCOR2
2	NCOR1
2	NCKAP5L
2	NCAPG
2	NBEA
2	NARS2
2	NAPEPLD
2	NAP1L2
2	NACA
2	MYOM1
2	MYO7B
2	MYO6
2	MYO5B
2	MYO5A
2	MYO1B
2	MYO18A
2	MYO15A
2	MYH2
2	MYBL1
2	MUT
2	MUL1
2	MUC6
2	MTA2
2	MSL3
2	MPDZ
2	MON2
2	MOCS2
2	MMS22L
2	MLL4
2	MEX3C
2	METTL16
2	METTL11B
2	METAP1D
2	MEGF8
2	MED13L
2	MED13
2	MECOM
2	MCTP1
2	MCM5
2	MCCC1
2	MAST4
2	MAST2
2	MAST1

2	MAP3K7
2	MAP2
2	MAP1A
2	MAN1A2
2	MAGI3
2	MACROD1
2	LYAR
2	LY75
2	LRRN1
2	LRRK2
2	LRRK1
2	LRP1
2	LRIG2
2	LRCH4
2	LPPR4
2	LPHN2
2	LOXHD1
2	LIN9
2	LIN7B
2	LGR4
2	LEO1
2	LCT
2	LAMA5
2	LAMA2
2	LAMA1
2	KRTAP6
2	KRTAP10
2	KPNA7
2	KNTC1
2	KLHL32
2	KLHL21
2	KIFC2
2	KIF21B
2	KIF15
2	KIAA1797
2	KIAA1239
2	KIAA0430
2	KIAA0368
2	KDR
2	KDM6B
2	KDM5D
2	KDELC1
2	KCNT2
2	KCNK4
2	KAT6B
2	KALRN
2	ITGB4
2	ITGB3BP
2	IQCB1
2	IPO7
2	INF2
2	IFI44L
2	IDE

2	IBTK
2	HSP90B1
2	HSF5
2	HNRNPK
2	HIVEP2
2	HIST3H3
2	HEY2
2	HERC4
2	HELZ
2	HEATR5A
2	HEATR3
2	HDX
2	HDAC4
2	HBS1L
2	HAS2
2	HACE1
2	GTPBP4
2	GRIN2B
2	GRIN1
2	GPR64
2	GPR22
2	GPR110
2	GPC4
2	GPAM
2	GMIP
2	GKAP1
2	GFAP
2	GDF6
2	GCFC1
2	GBP3
2	GABBR2
2	FUK
2	FUBP1
2	FTSJD2
2	FTSJ3
2	FRMPD4
2	FRMPD1
2	FNDC3A
2	FMNL3
2	FMNL2
2	FLNA
2	FHOD1
2	FH
2	FCRL3
2	FBXW9
2	FBXO38
2	FBXL19
2	FASN
2	FAM69B
2	FAM65C
2	FAM190B
2	FAM169A
2	FAM13B

2 EYS
2 EXOC1
2 ESD
2 EPS15
2 EPG5
2 EPB41L4B
2 EP300
2 EML5
2 ELOVL4
2 EIF3B
2 EIF2C3
2 EGR1
2 EEPD1
2 ECEL1
2 DUSP5
2 DSP
2 DRD4
2 DPYSL4
2 DPY19L3
2 DOCK10
2 DNMT3B
2 DNHD1
2 DNAJC13
2 DNAJA4
2 DNAH9
2 DNAH1
2 DLGAP1
2 DLD
2 DIP2A
2 DHX36
2 DFNB59
2 DEPDC5
2 DDX54
2 DDX5
2 DDX47
2 DDHD1
2 DAPK1
2 DACH2
2 CUL9
2 CUL5
2 CUL4B
2 CTTNBP2
2 CSDE1
2 CREBBP
2 CPVL
2 CPS1
2 CPNE5
2 COPA
2 COL28A1
2 COL22A1
2 COL17A1
2 CNTNAP2
2 CNTN3

2 CNTLN
2 CNKSR2
2 CNGB3
2 CLSTN2
2 CLIP1
2 CLCN6
2 CKB
2 CIT
2 CHKA
2 CHD8
2 CHD7
2 CHD4
2 CHD1
2 CEP350
2 CEP104
2 CELSR3
2 CELSR1
2 CDK5RAP1
2 CDHR4
2 CDH6
2 CDH5
2 CDH10
2 CDC14A
2 CD180
2 CCNT1
2 CCDC73
2 CCDC67
2 CCDC158
2 CCDC110
2 CC2D1A
2 CBFA2T3
2 CASKIN2
2 CAPZA2
2 CAMTA1
2 CAMSAP3
2 CAD
2 CACNA1G
2 CACHD1
2 CA8
2 C9orf5
2 C6orf186
2 C5
2 C4orf33
2 C20orf7
2 C1orf9
2 C11orf9
2 C11orf41
2 BUB1B
2 BTBD1
2 BSN
2 BROX
2 BMS1
2 BLM

2 BCORL1
2 BCKDHB
2 BBS5
2 AUTS2
2 ATXN1
2 ATP8A1
2 ATP11C
2 ATG10
2 ATF6
2 ASTN1
2 ASPM
2 ASH1L
2 ASCC3
2 ASAP1
2 ARL5A
2 ARID2
2 ARID1A
2 ARHGAP44
2 ARHGAP12
2 ARFGEF1
2 AP3B1
2 ANO7
2 ANKRD28
2 ANKHD1
2 ANK3
2 ALX4
2 ALS2
2 AHR
2 AHI1
2 AGPS
2 AGL
2 AGER
2 AGBL3
2 ADAMTS6
2 ADAMTS12
2 ACTN4
2 ACRC
2 ACOT11
2 ACIN1
2 ABCC9
2 ABCC6
2 ABCC2
2 ABCA9
2 ABCA1
1 ZZEF1
1 ZSWIM2
1 ZSCAN20
1 ZSCAN16
1 ZSCAN1
1 ZNRF3
1 ZNHIT6
1 ZNF93
1 ZNF92

1	ZNF90
1	ZNF879
1	ZNF845
1	ZNF836
1	ZNF830
1	ZNF83
1	ZNF823
1	ZNF816
1	ZNF81
1	ZNF804A
1	ZNF800
1	ZNF791
1	ZNF784
1	ZNF766
1	ZNF76
1	ZNF750
1	ZNF709
1	ZNF708
1	ZNF704
1	ZNF701
1	ZNF700
1	ZNF697
1	ZNF674
1	ZNF648
1	ZNF644
1	ZNF642
1	ZNF627
1	ZNF625
1	ZNF622
1	ZNF619
1	ZNF616
1	ZNF613
1	ZNF610
1	ZNF609
1	ZNF607
1	ZNF599
1	ZNF592
1	ZNF586
1	ZNF585A
1	ZNF570
1	ZNF554
1	ZNF551
1	ZNF548
1	ZNF541
1	ZNF532
1	ZNF530
1	ZNF521
1	ZNF496
1	ZNF484
1	ZNF449
1	ZNF439
1	ZNF433
1	ZNF398

1	ZNF362
1	ZNF354C
1	ZNF345
1	ZNF341
1	ZNF330
1	ZNF326
1	ZNF320
1	ZNF318
1	ZNF292
1	ZNF28
1	ZNF277
1	ZNF256
1	ZNF25
1	ZNF248
1	ZNF24
1	ZNF234
1	ZNF232
1	ZNF23
1	ZNF205
1	ZNF202
1	ZNF189
1	ZNF184
1	ZNF167
1	ZNF16
1	ZNF142
1	ZNF14
1	ZNF133
1	ZNF12
1	ZNF114
1	ZNF107
1	ZMYND8
1	ZMYM6
1	ZMYM1
1	ZMAT2
1	ZMAT1
1	ZKSCAN5
1	ZKSCAN3
1	ZIC3
1	ZFYVE26
1	ZFX
1	ZFR
1	ZFPM2
1	ZFP91
1	ZFP37
1	ZFP28
1	ZFP14
1	ZFP112
1	ZFP1
1	ZFAT
1	ZDHHC23
1	ZDHHC2
1	ZDHHC13
1	ZCRB1

1	ZCCHC4
1	ZC3HC1
1	ZC3H13
1	ZBTB8B
1	ZBTB49
1	ZBTB40
1	ZBTB39
1	ZBTB24
1	ZBTB1
1	ZBBX
1	ZADH2
1	YWHAZ
1	YWHAG
1	YSK4
1	YPEL3
1	YME1L1
1	YEATS2
1	XYLT2
1	XRN2
1	XRCC6
1	XPOT
1	XPO7
1	XPO4
1	XDH
1	XAB2
1	WWP1
1	WWC1
1	WSB1
1	WRAP73
1	WNT8B
1	WNT5B
1	WNT2B
1	WNK4
1	WNK1
1	WDTC1
1	WDR91
1	WDR67
1	WDR63
1	WDR5B
1	WDR49
1	WDR45L
1	WDR41
1	WDR38
1	WDR36
1	WDR3
1	WDR20
1	WDFY4
1	WASF2
1	WASF1
1	WAPAL
1	VWDE
1	VWA3B
1	VWA2

1 VTI1A
1 VPS52
1 VPS4B
1 VPS36
1 VPS33A
1 VLDLR
1 VIT
1 VIPR1
1 VGF
1 VCPIP1
1 VCP
1 VCL
1 VCAN
1 VAV3
1 VASN
1 UTP20
1 USP8
1 USP7
1 USP53
1 USP48
1 USP47
1 USP43
1 USP35
1 USP33
1 USP25
1 USP13
1 USH1C
1 USF1
1 UPK1B
1 UPF2
1 UNK
1 UNC13C
1 ULK4
1 UGT1A9
1 UGT1A8
1 UGT1A7
1 UGT1A6
1 UGT1A5
1 UGT1A4
1 UGT1A3
1 UGT1A10
1 UGT1A1
1 UGDH
1 UFL1
1 UCP1
1 UCHL3
1 UBTD1
1 UBR5
1 UBR2
1 UBQLN2
1 UBQLN1
1 UBE4B
1 UBE4A

1 UBE3A
1 UBE2E2
1 UBASH3B
1 UBAP2L
1 UBAP2
1 UACA
1 U2AF2
1 TYW5
1 TYR
1 TXNDC3
1 TUSC3
1 TUBGCP6
1 TUBGCP3
1 TUBD1
1 TUBA3C
1 TTLL5
1 TTI1
1 TTC8
1 TTC7B
1 TTC7A
1 TTC5
1 TTC37
1 TTC27
1 TTC21B
1 TTC21A
1 TTC12
1 TSR1
1 TSPAN31
1 TSN
1 TSKU
1 TSHZ2
1 TSHR
1 TSC2
1 TRPS1
1 TRPM7
1 TRPM4
1 TRPC4AP
1 TRMT5
1 TRMT11
1 TRIT1
1 TRIP12
1 TRIP11
1 TRIM8
1 TRIM63
1 TRIM41
1 TRIM3
1 TRIM24
1 TRIM17
1 TRIM13
1 TRERF1
1 TRDMT1
1 TRAPPC9
1 TRAPPC5

1 TRAPPC3
1 TPM3
1 TP73
1 TP53I13
1 TOP1
1 TOMM70A
1 TOM1
1 TOE1
1 TNS1
1 TNRC6B
1 TNR
1 TNPO3
1 TNNT2
1 TNN
1 TNKS2
1 TNKS
1 TNK2
1 TNFSF8
1 TNFAIP8L3
1 TNF
1 TMTC2
1 TMPRSS9
1 TMF1
1 TMEM8A
1 TMEM87B
1 TMEM85
1 TMEM63B
1 TMEM63A
1 TMEM41B
1 TMEM26
1 TMEM200C
1 TMEM200A
1 TMEM19
1 TMEM165
1 TMEM151B
1 TMEM135
1 TMEM132B
1 TMEM131
1 TMEM126B
1 TMEM108
1 TMEM106A
1 TMEM104
1 TMED5
1 TMC8
1 TMC1
1 TM7SF3
1 TM6SF1
1 TLR10
1 TLN2
1 TLN1
1 TLL1
1 TLK2
1 TLK1

1	TKTL1
1	TIGD3
1	TIAM1
1	THUMPD1
1	THSD7A
1	THOC2
1	THOC1
1	THEM5
1	TGM4
1	TGFBR3
1	TGFBR2
1	TGFB1I1
1	TGFB1
1	TGDS
1	TFR2
1	TFEC
1	TFDP2
1	TFB2M
1	TF
1	TEX2
1	TEX15
1	TEX14
1	TESK1
1	TERF1
1	TEKT3
1	TEK
1	TECRL
1	TECPR1
1	TEAD2
1	TDRD9
1	TDRD7
1	TDRD12
1	TDP1
1	TCP11L2
1	TCIRG1
1	TCHP
1	TCHH
1	TCF4
1	TCEANC
1	TBX3
1	TBX2
1	TBPL2
1	TBL3
1	TBCK
1	TBCD
1	TBCC
1	TBCB
1	TBC1D9B
1	TBC1D8B
1	TBC1D7
1	TBC1D5
1	TBC1D25
1	TBC1D23

1	TBC1D19
1	TBC1D17
1	TBC1D12
1	TARBP1
1	TANC1
1	TAGAP
1	TAF7L
1	TAF6L
1	TAF4B
1	TAF4
1	TAF15
1	TAF1
1	TADA2A
1	TACSTD2
1	TACO1
1	TAB2
1	TAAR6
1	TAAR2
1	T
1	SYTL2
1	SYNJ2BP
1	SYNGAP1
1	SYMPK
1	SYCP1
1	SWT1
1	SWAP70
1	SVOPL
1	SUV39H2
1	SUSD2
1	SUSD1
1	SUPT6H
1	SUN3
1	SULF1
1	SUGP1
1	SUFU
1	SUCLA2
1	STXBP5L
1	STXBP4
1	STX19
1	STT3B
1	STRN3
1	STOX2
1	STMN3
1	STK38
1	STK3
1	STAU2
1	STAT6
1	STAT4
1	STARD6
1	STARD13
1	STAG2
1	STAG1
1	STAB1

1	ST8SIA3
1	ST7L
1	SSFA2
1	SSBP4
1	SSBP3
1	SRRT
1	SRRM1
1	SRPK2
1	SRPK1
1	SRP54
1	SRP19
1	SRMS
1	SRCIN1
1	SRA1
1	SQRDL
1	SPTBN1
1	SPRYD7
1	SPPL3
1	SPO11
1	SPNS1
1	SPICE1
1	SPHK2
1	SPHK1
1	SPG20
1	SPEN
1	SPEF2
1	SPECC1
1	SPDEF
1	SPATA6
1	SPATA5
1	SPATA13
1	SPARCL1
1	SPAG6
1	SPAG5
1	SP100
1	SP1
1	SOX17
1	SOX11
1	SOST
1	SORCS1
1	SORBS3
1	SORBS2
1	SORBS1
1	SOCS4
1	SNX8
1	SNX6
1	SNX5
1	SNX4
1	SNX2
1	SNX10
1	SNRPE
1	SNRPD1
1	SNRPA1

1	SNRNP200
1	SNAI3
1	SNAI2
1	SMU1
1	SMTN
1	SMPD3
1	SMG6
1	SMC6
1	SMC3
1	SMARCC1
1	SMARCA5
1	SMAP2
1	SMAGP
1	SMAD6
1	SLITRK6
1	SLITRK5
1	SLITRK3
1	SLITRK2
1	SLIT2
1	SLCO2B1
1	SLCO1C1
1	SLC9A7
1	SLC9A6
1	SLC9A5
1	SLC9A4
1	SLC9A2
1	SLC9A10
1	SLC7A4
1	SLC6A3
1	SLC6A15
1	SLC6A14
1	SLC5A7
1	SLC4A9
1	SLC44A5
1	SLC44A2
1	SLC44A1
1	SLC38A2
1	SLC36A4
1	SLC35F4
1	SLC35F3
1	SLC35D2
1	SLC35B2
1	SLC32A1
1	SLC30A9
1	SLC30A10
1	SLC2A6
1	SLC2A4RG
1	SLC2A10
1	SLC29A2
1	SLC28A1
1	SLC26A6
1	SLC26A5
1	SLC26A3

1 SLC25A48
1 SLC25A44
1 SLC25A36
1 SLC25A33
1 SLC25A31
1 SLC25A29
1 SLC25A16
1 SLC25A14
1 SLC24A1
1 SLC23A3
1 SLC22A15
1 SLC22A1
1 SLC18A3
1 SLC18A1
1 SLC16A9
1 SLC16A4
1 SLC13A1
1 SLC12A3
1 SLC12A2
1 SKIV2L2
1 SKIV2L
1 SKIL
1 SIX3
1 SIRT5
1 SIPA1L3
1 SIPA1
1 SIN3B
1 SIN3A
1 SIK3
1 SIDT2
1 SIAH3
1 SIAH2
1 SIAE
1 SHROOM3
1 SHROOM2
1 SHPRH
1 SHOC2
1 SHMT2
1 SHKBP1
1 SHANK3
1 SH3GLB2
1 SH3GLB1
1 SH2D3C
1 SH2B3
1 SGK223
1 SGK196
1 SGK1
1 SGCG
1 SFSWAP
1 SFRP1
1 SFMBT2
1 SF1
1 SEZ6L

1 SETX
1 SETD3
1 SETBP1
1 SESTD1
1 SESN1
1 SERPINF2
1 SERPIND1
1 SERPINB2
1 SERPINB13
1 SERINC1
1 SERF2
1 SEPT8
1 SEPT4
1 SEPT12
1 SENP2
1 SEMA6D
1 SEMA6B
1 SEMA4F
1 SEMA3G
1 SEMA3F
1 SEMA3E
1 SEMA3C
1 SEMA3A
1 SEL1L3
1 SEL1L2
1 SEH1L
1 SEC62
1 SEC61A2
1 SEC24B
1 SEC24A
1 SEC13
1 SDC2
1 SCYL3
1 SCUBE3
1 SCRN1
1 SCNN1D
1 SCN8A
1 SCN7A
1 SCN3A
1 SCN10A
1 SCFD2
1 SCARF2
1 SCARA3
1 SCAMP3
1 SCAF11
1 SBNO1
1 SAMSN1
1 SAMD9L
1 SAMD14
1 SAMD10
1 S100PBP
1 RXFP3
1 RWDD2A

1	RUVBL2
1	RUSC2
1	RUSC1
1	RUNDC3B
1	RUFY2
1	RTN3
1	RTN1
1	RTCD1
1	RSRC2
1	RSRC1
1	RSPRY1
1	RRP12
1	RPS6KA4
1	RPS2
1	RPS13
1	RPS12
1	RPRD2
1	RPRD1A
1	RPL9
1	RPL4
1	RPL28
1	RPL14
1	RPGRIP1L
1	RP2
1	RORC
1	RORA
1	ROCK2
1	ROCK1
1	RNF43
1	RNF31
1	RNF215
1	RNF170
1	RNF165
1	RNF150
1	RNF149
1	RNF130
1	RNF128
1	RNF111
1	RNF10
1	RIT2
1	RIMS1
1	RIMBP2
1	RHPN1
1	RHOBTB3
1	RHBDF1
1	RHBDD1
1	RGS11
1	RGL1
1	RG9MTD2
1	RFXAP
1	RFX6
1	RFX3
1	RFX1

1 RFFL
1 RFC3
1 REV1
1 RETN
1 RET
1 RERE
1 REP15
1 RELL2
1 RELB
1 RECQL
1 RDX
1 RDM1
1 RCN1
1 RC3H1
1 RBPJ
1 RBMS2
1 RBM5
1 RBM39
1 RBM22
1 RBM19
1 RBM18
1 RBL1
1 RBFOX2
1 RBFOX1
1 RB1
1 RAX2
1 RASSF1
1 RASGRP4
1 RASGRP3
1 RASGRF1
1 RASAL2
1 RARS2
1 RAPGEF6
1 RAPGEF1
1 RAP2C
1 RAP1GDS1
1 RANBP17
1 RANBP10
1 RALGPS2
1 RALGPS1
1 RALA
1 RAI1
1 RAD54B
1 RAD50
1 RAD21L1
1 RACGAP1
1 RAB8B
1 RAB3GAP1
1 RAB23
1 RAB11FIP5
1 R3HDM2
1 QTRT1
1 QSOX2

1 QRICH2
1 QRFPR
1 QARS
1 PYGO2
1 PYGM
1 PYCR1
1 PWWP2B
1 PVRL3
1 PVRL2
1 PUS7L
1 PUS10
1 PTRF
1 PTPRZ1
1 PTPRT
1 PTPRK
1 PTPRG
1 PTPRF
1 PTPRB
1 PTPRA
1 PTPN4
1 PTPN22
1 PTPN13
1 PTPN11
1 PTPDC1
1 PTP4A1
1 PTMA
1 PTK7
1 PTGR2
1 PTGER1
1 PTGDR
1 PTCHD2
1 PTCHD1
1 PTCH2
1 PTCD1
1 PSTK
1 PSMD2
1 PSMB8
1 PSMB10
1 PSMA1
1 PSKH2
1 PSKH1
1 PRUNE2
1 PRSS53
1 PRSS16
1 PRSS12
1 PRRG4
1 PRRC1
1 PRR7
1 PRR16
1 PRR14
1 PRPF4
1 PRPF31
1 PRPF19

1 ProSAPiP1
1 PRMT10
1 PRKG2
1 PRKG1
1 PRKCE
1 PRKAR2B
1 PRICKLE2
1 PRICKLE1
1 PRG4
1 PRDX5
1 PRDM6
1 PRDM5
1 PRDM4
1 PRDM13
1 PRCP
1 PRC1
1 PPTC7
1 PPP6R1
1 PPP4R4
1 PPP4R2
1 PPP3CC
1 PPP1R3F
1 PPP1R3C
1 PPP1R15B
1 PPP1R15A
1 PPP1R13B
1 PPM1N
1 PPM1L
1 PPM1E
1 PPM1B
1 PPIP5K2
1 PPHLN1
1 PPFIBP2
1 PPEF2
1 PPA2
1 POU3F4
1 POU3F3
1 POU3F2
1 POSTN
1 PORCN
1 POPDC3
1 POMC
1 POLR3G
1 POLR1D
1 POLR1A
1 POGZ
1 POF1B
1 PODXL2
1 POC1B
1 PNPT1
1 PNPLA7
1 PNPLA4
1 PNPLA2

1 PNCK
1 PMPCB
1 PMPCA
1 PMP2
1 PM20D2
1 PM20D1
1 PLXND1
1 PLXNB3
1 PLXNB2
1 PLXNA3
1 PLTP
1 PLSCR4
1 PLS1
1 PLIN2
1 PLIN1
1 PLEKHH1
1 PLEKHA8
1 PLEKHA7
1 PLEKHA6
1 PLEKHA4
1 PLDN
1 PLCZ1
1 PLCL2
1 PLCD4
1 PLCB1
1 PLA2G4A
1 PKP4
1 PKP2
1 PKP1
1 PKN3
1 PKN2
1 PKMYT1
1 PKD1L1
1 PIWIL3
1 PIWIL2
1 PIPOX
1 PION
1 PIK3AP1
1 PIGU
1 PIGQ
1 PIGK
1 PHTF1
1 PHPT1
1 PHF8
1 PHF3
1 PHF20
1 PHF2
1 PHF15
1 PHF14
1 PHC3
1 PHC2
1 PGRMC1
1 PGR

1 PGM3
1 PGM2L1
1 PGAP1
1 PFKFB3
1 PEX1
1 PER3
1 PELI3
1 PEG3
1 PEAR1
1 PEAK1
1 PDZRN4
1 PDXDC1
1 PDS5A
1 PDLIM5
1 PDLIM4
1 PDHA1
1 PDGFRL
1 PDGFRA
1 PDE8B
1 PDE6C
1 PDE12
1 PDE11A
1 PDCL3
1 PDCD6IP
1 PDCD4
1 PDC
1 PCSK9
1 PCSK4
1 PCP2
1 PCOLCE2
1 PCNXL2
1 PCNP
1 PCDHGC3
1 PCDHA3
1 PCDH9
1 PCDH17
1 PCDH11X
1 PCBP2
1 PCBD2
1 PC
1 PBRM1
1 PAX6
1 PATL2
1 PATL1
1 PARP9
1 PARP6
1 PARP2
1 PARL
1 PARD6G
1 PARD3B
1 PARD3
1 PAPSS1
1 PAPPA2

1 PAPPA
1 PAPD4
1 PADI2
1 PACSIN1
1 PACS2
1 PABPN1
1 P4HA3
1 P4HA1
1 P2RY10
1 OXA1L
1 OSTF1
1 OSGEPL1
1 OSBPL9
1 OSBPL1A
1 OSBPL11
1 OS9
1 ORC4
1 ORC2
1 OR8K5
1 OR6K2
1 OR6B1
1 OR5P2
1 OR5F1
1 OR5B17
1 OR52L1
1 OR52E2
1 OR51F2
1 OR2M4
1 OR1J2
1 OR13C4
1 OR10G7
1 OR10A7
1 OPRM1
1 OPRL1
1 OPN3
1 ONECUT2
1 OLIG3
1 OLIG2
1 OIT3
1 OGFRL1
1 OCA2
1 OBSL1
1 NXPH2
1 NXNL2
1 NWD1
1 NUP214
1 NUP160
1 NUP133
1 NUMA1
1 NUFIP1
1 NUCKS1
1 NUBP1
1 NTRK1

1 NT5C2
1 NSUN2
1 NSMCE4A
1 NSL1
1 NRXN3
1 NRXN1
1 NRTN
1 NRF1
1 NRCAM
1 NRBP1
1 NR3C2
1 NR3C1
1 NR2C1
1 NPS
1 NPR2
1 NPM1
1 NPLOC4
1 NPL
1 NPC1
1 NPBWR2
1 NPAS2
1 NPAS1
1 NOX3
1 NOTUM
1 NOS3
1 NOP56
1 NOP14
1 NONO
1 NOL8
1 NOL6
1 NOC3L
1 NMUR2
1 NMNAT1
1 NM_001164508
1 NM_001164507
1 NM_001081491
1 NLN
1 NLK
1 NLGN3
1 NLGN2
1 NKRF
1 NIT1
1 NISCH
1 NIPA1
1 NINL
1 NID2
1 NID1
1 NHS
1 NHLRC1
1 NHLH2
1 NHEJ1
1 NGLY1
1 NFXL1

1 NFX1
1 NFRKB
1 NFKBIZ
1 NFKB1
1 NFE2L2
1 NFATC2
1 NFAT5
1 NFASC
1 NF1
1 NEXN
1 NEU2
1 NETO2
1 NEMF
1 NELL2
1 NEK7
1 NEK5
1 NEK1
1 NECAB1
1 NDUFAF1
1 NDUFA3
1 NDNL2
1 NDNF
1 NDC80
1 NCOA7
1 NCOA3
1 NCLN
1 NCKAP1L
1 NCAPH
1 NCAPD2
1 NCAM2
1 NBEAL2
1 NAT14
1 NARF
1 NAP1L4
1 NANS
1 NANOS1
1 NALCN
1 NACC1
1 NAALAD2
1 NAA16
1 N4BP2L2
1 N4BP2L1
1 MYSM1
1 MYRIP
1 MYPN
1 MYO9A
1 MYO3B
1 MYO1F
1 MYO1E
1 MYO1D
1 MYO1C
1 MYLIP
1 MYH9

1 MYH8
1 MYH3
1 MYH14
1 MYH13
1 MYH11
1 MYH1
1 MYC
1 MYBPC2
1 MYBPC1
1 MYB
1 MYADML2
1 MX2
1 MUC7
1 MUC4
1 MTRF1L
1 MTR
1 MTMR8
1 MTMR4
1 MTMR12
1 MTM1
1 MTHFD1L
1 MTERFD2
1 MTA1
1 MST4
1 MST1R
1 MSH6
1 MSH2
1 MRPS35
1 MRPS27
1 MRPL38
1 MRPL22
1 MRE11A
1 MPP5
1 MPP4
1 MPHOSPH9
1 MPHOSPH10
1 MORC3
1 MORC1
1 MOGAT1
1 MOG
1 MOBKL2B
1 MNDA
1 MMS19
1 MMP24
1 MLNR
1 MLLT4
1 MLLT3
1 MLLT10
1 MLL5
1 MLL3
1 MLIP
1 MLF1IP
1 MKL1

1 MKI67
1 MIS18BP1
1 MIOS
1 MICALCL
1 MICAL2
1 MIA3
1 MGRN1
1 MGAT4A
1 MGAT3
1 MGAM
1 MFSD6L
1 MFSD4
1 MFHAS1
1 MFF
1 MFAP5
1 METTL5
1 METTL3
1 METTL18
1 MET
1 MEOX2
1 MELK
1 MEI1
1 MEGF11
1 MEF2D
1 MED8
1 MED6
1 MED21
1 MED15
1 MED14
1 MECR
1 MECP2
1 MDM2
1 MCTP2
1 MCM7
1 MCM3AP
1 MCF2L
1 MCAT
1 MCAM
1 MC3R
1 MC2R
1 MBOAT7
1 MBNL3
1 MAZ
1 MAST3
1 MARK2
1 MARK1
1 MARCH7
1 MARCH1
1 MAPK8IP1
1 MAP7D2
1 MAP7D1
1 MAP3K8
1 MAP3K6

1 MAP3K13
1 MAP3K11
1 MAP3K10
1 MAP2K6
1 MAP2K5
1 MAP1B
1 MAOB
1 MAN2B1
1 MAMLD1
1 MALT1
1 MAGT1
1 MAGI2
1 MAGI1
1 MAGED2
1 MAD1L1
1 LZTS1
1 LZTR1
1 LYSMD4
1 LYSMD2
1 LTC4S
1 LTBP3
1 LTB4R
1 LTB
1 LSS
1 LSR
1 LSM14A
1 LSM12
1 LRRTM1
1 LRRN3
1 LRRIQ1
1 LRRC71
1 LRRC7
1 LRRC58
1 LRRC36
1 LRRC31
1 LRRC27
1 LRRC26
1 LRP8
1 LRP6
1 LRP3
1 LRP12
1 LRIG1
1 LRFN5
1 LPO
1 LPHN1
1 LPGAT1
1 LPCAT4
1 LPCAT3
1 LPCAT2
1 LONRF3
1 LMX1A
1 LMTK3
1 LMNB1

1 LMF1
1 LMBRD1
1 LLGL1
1 LIPT2
1 LIPF
1 LIN54
1 LIMD1
1 LIMCH1
1 LIMA1
1 LIG4
1 LIFR
1 LHCGR
1 LGI1
1 LGALSL
1 LGALS8
1 LFNG
1 LEPREL4
1 LDB3
1 LCP2
1 LBR
1 LARP4
1 LAPTM4B
1 LAMC3
1 LAMC2
1 LAMB4
1 LACTB2
1 L3MBTL4
1 KYNU
1 KY
1 KRTCAP3
1 KRTAP1
1 KRT9
1 KRT84
1 KRT82
1 KRT8
1 KRT5
1 KRT40
1 KRT33A
1 KRIT1
1 KPNA6
1 KPNA5
1 KPNA3
1 KNG1
1 KMO
1 KLRG2
1 KLRAQ1
1 KLHL8
1 KLHL31
1 KLHL3
1 KLHL17
1 KLHL14
1 KLHL11
1 KLHL10

1	KLHL1
1	KLHDC2
1	KLF5
1	KLF2
1	KLC3
1	KIFAP3
1	KIF7
1	KIF5B
1	KIF4A
1	KIF3A
1	KIF26B
1	KIF26A
1	KIF23
1	KIF22
1	KIF1A
1	KIF17
1	KIF16B
1	KIF13A
1	KIF11
1	KIAA2022
1	KIAA2018
1	KIAA1804
1	KIAA1731
1	KIAA1530
1	KIAA1524
1	KIAA1468
1	KIAA1462
1	KIAA1407
1	KIAA1324L
1	KIAA1279
1	KIAA1191
1	KIAA1143
1	KIAA1045
1	KIAA1009
1	KIAA0895
1	KIAA0889
1	KIAA0748
1	KIAA0556
1	KIAA0494
1	KIAA0355
1	KIAA0319L
1	KIAA0226
1	KIAA0196
1	KIAA0195
1	KIAA0020
1	KHSRP
1	KHDRBS2
1	KHDRBS1
1	KEL
1	KDM5C
1	KDM5A
1	KDM3A
1	KDM1A

1 KDELC2
1 KCTD3
1 KCTD18
1 KCNV2
1 KCNRG
1 KCNQ2
1 KCNN2
1 KCNMA1
1 KCNK16
1 KCNK10
1 KCNJ1
1 KCNIP4
1 KCNH8
1 KCNH7
1 KCNH5
1 KCNH4
1 KCNH1
1 KCNG4
1 KCNG2
1 KCNA4
1 KCNA1
1 KBTBD11
1 KAT6A
1 KANK2
1 KANK1
1 JPH2
1 JOSD2
1 JAKMIP2
1 JAK3
1 IWS1
1 IVNS1ABP
1 ITSN2
1 ITSN1
1 ITPR3
1 ITPR2
1 ITM2B
1 ITGB1
1 ITGAV
1 ITGA8
1 ITGA2
1 ITFG3
1 IRX6
1 IRS4
1 IRS2
1 IRGQ
1 IRF2BP2
1 IREB2
1 IQSEC3
1 IQGAP3
1 IQGAP2
1 IQGAP1
1 IQCA1
1 IPO8

1 IPMK
1 IPCEF1
1 INTS6
1 INTS1
1 INSM2
1 INPP5B
1 INPP4A
1 INO80D
1 INO80C
1 INO80B
1 INO80
1 INADL
1 IMPACT
1 IMP5
1 IMMT
1 ILVBL
1 ILF3
1 IL6ST
1 IL6R
1 IL2RG
1 IL27RA
1 IL22RA1
1 IL12RB2
1 IKZF5
1 IGSF8
1 IGFBP3
1 IGFALS
1 IGF2R
1 IGF2BP3
1 IGF1R
1 IFT88
1 IFT80
1 IFFO2
1 ICA1L
1 IARS2
1 HTT
1 HTRA4
1 HTR2C
1 HSPH1
1 HSPE1
1 HSPD1
1 HSPB11
1 HSPA9
1 HSPA12A
1 HSD17B3
1 HSD17B10
1 HS6ST3
1 HS6ST2
1 HS3ST5
1 HS3ST1
1 HPS5
1 HPS3
1 HPRT1

1 HPDL
1 HP1BP3
1 HOXB13
1 HOXA3
1 HOMER3
1 HNRNPM
1 HNRNPH1
1 HNRNPA2B1
1 HNF4G
1 HMX3
1 HMGCS1
1 HMGCR
1 HMGB1
1 HMG20B
1 HM13
1 HKR1
1 HIST1H2AH
1 HIPK3
1 HIPK1
1 HINT2
1 HINFP
1 HIF3A
1 HIC1
1 HIAT1
1 HHIPL1
1 HGFAC
1 HGF
1 HFM1
1 HFE
1 HES4
1 HERPUD2
1 HERC3
1 HEPHL1
1 HEPH
1 HELQ
1 HELLS
1 HECW2
1 HECW1
1 HECTD2
1 HEATR8
1 HEATR7B2
1 HDLBP
1 HDAC8
1 HDAC7
1 HDAC3
1 HDAC10
1 HCRTR1
1 HCN1
1 HCFC2
1 HCFC1
1 HAS3
1 HAND1
1 HAL

1 H3F3A
1 GYLTL1B
1 GXYLT1
1 GUCY1B3
1 GTF3C4
1 GTF3C3
1 GTF3C1
1 GTF2IRD1
1 GTF2F2
1 GTF2F1
1 GSTM3
1 GSTCD
1 GSTA5
1 GSPT2
1 GSDMA
1 GRWD1
1 GRRP1
1 GRM1
1 GRK5
1 GRIN3B
1 GRIN3A
1 GRIK5
1 GRIK1
1 GREB1
1 GRB2
1 GRB14
1 GRAMD1A
1 GPX2
1 GPT2
1 GPSM2
1 GPRC6A
1 GPRC5B
1 GPR88
1 GPR78
1 GPR75
1 GPR56
1 GPR4
1 GPR180
1 GPR179
1 GPR161
1 GPR157
1 GPR156
1 GPR155
1 GPR137B
1 GPR137
1 GPR126
1 GPCPD1
1 GPC5
1 GPC3
1 GPBP1L1
1 GPBP1
1 GPBAR1
1 GPATCH2

1 GPATCH1
1 GPAA1
1 GOT2
1 GOPC
1 GOLPH3L
1 GOLGA7B
1 GOLGA5
1 GOLGA4
1 GNS
1 GNPTAB
1 GNL1
1 GNB2
1 GNAI3
1 GMFG
1 GLTSCR2
1 GLTPD1
1 GLT8D1
1 GLO1
1 GLI2
1 GLDN
1 GK2
1 GK
1 GJA5
1 GIPR
1 GINS4
1 GINS3
1 GIGYF2
1 GHR
1 GGPS1
1 GFOD2
1 GFM2
1 GET4
1 GEMIN5
1 GDF11
1 GDF1
1 GDA
1 GCLM
1 GCDH
1 GBF1
1 GATSL3
1 GARNL3
1 GANAB
1 GALNT7
1 GALM
1 GALE
1 GAL3ST4
1 GAL3ST1
1 GADD45B
1 GAD1
1 GABRG2
1 GABRB3
1 GABPA
1 FZD6

1 FYCO1
1 FUZ
1 FUT11
1 FUS
1 FURIN
1 FTSJD1
1 FSTL5
1 FRMD8
1 FRMD4A
1 FRMD1
1 FOXP2
1 FOXO3
1 FOXN4
1 FOXN2
1 FOXL2
1 FOXK1
1 FOXG1
1 FOXF2
1 FOXE3
1 FOXC1
1 FOSL2
1 FNTA
1 FNIP1
1 FNDC1
1 FN3K
1 FN1
1 FLRT3
1 FLNC
1 FLNB
1 FLII
1 FLI1
1 FKTN
1 FKBP15
1 FKBP14
1 FJX1
1 FILIP1
1 FIG4
1 FHDC1
1 FGL1
1 FGFR4
1 FGFR3
1 FGFR2
1 FGF3
1 FGF14
1 FGF13
1 FGD5
1 FERMT2
1 FER
1 FCRL4
1 FCGRT
1 FBXO46
1 FBXO4
1 FBXO31

1 FBXO30
1 FBXO16
1 FBXO15
1 FBXL5
1 FBXL12
1 FBRS
1 FBN3
1 FBN2
1 FAT2
1 FASTKD2
1 FASTKD1
1 FARSB
1 FANCC
1 FAN1
1 FAM91A1
1 FAM83B
1 FAM82B
1 FAM82A2
1 FAM82A1
1 FAM70A
1 FAM63B
1 FAM60A
1 FAM5C
1 FAM5B
1 FAM54A
1 FAM46D
1 FAM46C
1 FAM46B
1 FAM40B
1 FAM20A
1 FAM188B
1 FAM184A
1 FAM172A
1 FAM171B
1 FAM166A
1 FAM150B
1 FAM135A
1 FAM126A
1 FAM114A1
1 FAM113A
1 FAM109B
1 FAM108B1
1 FAM103A1
1 FAM102B
1 FAM102A
1 FAAH2
1 F5
1 EYA3
1 EXT2
1 EXOG
1 EXOC5
1 EVI5
1 EVC2

1 ETNK2
1 ETNK1
1 ESYT2
1 ESRP2
1 ESCO1
1 ERP29
1 ERO1LB
1 ERN2
1 ERMP1
1 ERMN
1 ERLIN1
1 ERGIC2
1 ERGIC1
1 ERCC5
1 ERCC4
1 ERC2
1 ERBB2IP
1 ERBB2
1 EPS8
1 EPRS
1 EPOR
1 EPM2A
1 EPHX2
1 EPHA5
1 EPHA4
1 EPHA10
1 EPB41L5
1 EPB41L3
1 EPB41L2
1 EPB41
1 ENTPD7
1 ENPP3
1 ENPP2
1 ENGASE
1 ENAM
1 ENAH
1 EML4
1 EML3
1 ELP3
1 ELMO3
1 ELF1
1 ELAVL3
1 EIF5
1 EIF4G2
1 EIF4ENIF1
1 EIF3D
1 EIF3A
1 EIF2C1
1 EIF2AK3
1 EIF2AK2
1 EIF2AK1
1 EIF2A
1 EHMT2

1	EHD2
1	EGFR
1	EGFLAM
1	EFTUD2
1	EFR3B
1	EFR3A
1	EFNA5
1	EFNA3
1	EFHA1
1	EFCAB6
1	EEFSEC
1	EDEM3
1	EDEM1
1	EDC4
1	ECT2L
1	ECE1
1	EBF1
1	E2F5
1	DZIP1
1	DYSF
1	DYRK1A
1	DYNC1I2
1	DYNC1H1
1	DVL2
1	DUSP2
1	DUOX2
1	DSG4
1	DSG2
1	DSCAML1
1	DSCAM
1	DROSHA
1	DRGX
1	DRG1
1	DQX1
1	DPY19L4
1	DPP10
1	DPM1
1	DOPEY1
1	DOK7
1	DOK5
1	DOK4
1	DOHH
1	DOCK4
1	DOCK3
1	DNTT
1	DNMT1
1	DNM1L
1	DNASE2B
1	DNAJC6
1	DNAJC25
1	DNAJC16
1	DNAJC15
1	DNAJB14

1 DNAJA3
1 DNAH6
1 DNAH10
1 DNA2
1 DMWD
1 DMRTA1
1 DMRT2
1 DMRT1
1 DMPK
1 DMC1
1 DLL4
1 DLL3
1 DLGAP5
1 DLG2
1 DLG1
1 DLEC1
1 DLAT
1 DKK3
1 DKC1
1 DIS3L
1 DIRC2
1 DIP2C
1 DIMT1
1 DICER1
1 DIAPH3
1 DIAPH2
1 DHX38
1 DHX35
1 DHX34
1 DHX16
1 DHX15
1 DHRS13
1 DGKB
1 DGKA
1 DGCR8
1 DGCR2
1 DFNB31
1 DEPDC7
1 DEPDC1B
1 DENND5A
1 DENND4C
1 DENND4B
1 DENND4A
1 DENND3
1 DENND2C
1 DENND1C
1 DENND1A
1 DEF6
1 DDX6
1 DDX4
1 DDX3X
1 DDX24
1 DDX23

1 DDX20
1 DDX10
1 DDX1
1 DDT
1 DDI2
1 DCTN1
1 DCLRE1C
1 DCLRE1A
1 DCLK1
1 DCHS2
1 DCHS1
1 DCDC2B
1 DCDC1
1 DCC
1 DCAF6
1 DCAF5
1 DCAF13
1 DBF4B
1 DAXX
1 DARS2
1 DAPK3
1 DAB1
1 DAAM1
1 CYP7B1
1 CYP4X1
1 CYP39A1
1 CYP2R1
1 CYP2C9
1 CYP20A1
1 CYLD
1 CYB5RL
1 CYB5D2
1 CX3CR1
1 CWH43
1 CWF19L1
1 CWC27
1 CWC22
1 CUZD1
1 CUL3
1 CTU2
1 CTNND2
1 CTNND1
1 CTNNBL1
1 CTNNAL1
1 CTNNA3
1 CTNNA2
1 CTNNA1
1 CTLA4
1 CTHRC1
1 CTH
1 CTBS
1 CSTF2T
1 CSTF1

1 CSRNP2
1 CSMD2
1 CSGALNACT2
1 CSE1L
1 CRYBA1
1 CRTC1
1 CRTAP
1 CROT
1 CROCC
1 CRNKL1
1 CRIM1
1 CREM
1 CRB1
1 CRAMP1L
1 CPSF2
1 CPNE1
1 CPM
1 CPLX1
1 CPEB3
1 CPD
1 CP
1 COQ10B
1 COMP
1 COMMD4
1 COLEC12
1 COL9A2
1 COL9A1
1 COL8A2
1 COL6A6
1 COL5A3
1 COL4A5
1 COL4A3BP
1 COL2A1
1 COL23A1
1 COL1A2
1 COL15A1
1 COG8
1 COG2
1 COG1
1 COBLL1
1 CNTN6
1 CNTN2
1 CNOT7
1 CNOT6
1 CNOT3
1 CNN1
1 CMYA5
1 CLTCL1
1 CLSTN3
1 CLRN1
1 CLPTM1L
1 CLK1
1 CLIP2

1 CLINT1
1 CLDN4
1 CKM
1 CKAP5
1 CIRH1A
1 CILP2
1 CIITA
1 CIC
1 CIAPIN1
1 CHTF18
1 CHSY3
1 CHST8
1 CHST3
1 CHST2
1 CHRNG
1 CHRNB1
1 CHRNA10
1 CHPF2
1 CHID1
1 CHIC1
1 CHEK2
1 CHEK1
1 CHD1L
1 CHAT
1 CHAF1B
1 CGN
1 CGGBP1
1 CFDP1
1 CEPT1
1 CEP97
1 CEP70
1 CEP57L1
1 CEP250
1 CEP128
1 CEP112
1 CENPB
1 CELSR2
1 CELF3
1 CELF2
1 CECR6
1 CEBPZ
1 CDON
1 CDKN1C
1 CDKL1
1 CDK7
1 CDK5R1
1 CDK20
1 CDK19
1 CDK17
1 CDK12
1 CDHR1
1 CDH9
1 CDH12

1 CDH1
1 CDC6
1 CDC45
1 CDC42BPB
1 CDC42BPA
1 CDC37L1
1 CDC23
1 CD96
1 CD200R1
1 CD163
1 CCT7
1 CCT5
1 CCT3
1 CCNL2
1 CCNI2
1 CCNI
1 CCNC
1 CCDC97
1 CCDC91
1 CCDC88B
1 CCDC88A
1 CCDC82
1 CCDC68
1 CCDC66
1 CCDC39
1 CCDC28B
1 CCDC165
1 CCDC157
1 CCDC15
1 CCDC142
1 CCDC141
1 CCDC125
1 CCDC117
1 CCDC104
1 CCDC102A
1 CC2D2A
1 CBR1
1 CBLL1
1 CBLC
1 CBL
1 CBFB
1 CAT
1 CASP2
1 CASKIN1
1 CASD1
1 CASC5
1 CARS2
1 CAPRIN2
1 CAPRIN1
1 CAPN7
1 CAPN2
1 CAP1
1 CAND2

1	CAND1
1	CAMTA2
1	CAMSAP2
1	CAMK2A
1	CAMK1G
1	CALR
1	CALML5
1	CALHM1
1	CALD1
1	CALCOCO2
1	CALCB
1	CACNB2
1	CACNB1
1	CACNA1D
1	CAB39
1	CA7
1	C9orf93
1	C9orf9
1	C9orf85
1	C9orf84
1	C9orf78
1	C9orf21
1	C9orf174
1	C9orf172
1	C9orf102
1	C9orf100
1	C8orf84
1	C7orf58
1	C7orf49
1	C7
1	C6orf89
1	C6orf203
1	C6orf145
1	C6orf136
1	C6orf103
1	C5orf54
1	C5orf51
1	C4orf14
1	C2orf69
1	C2orf66
1	C22orf28
1	C22orf26
1	C22orf13
1	C20orf94
1	C20orf194
1	C20orf12
1	C1QL4
1	C1orf93
1	C1orf55
1	C1orf49
1	C1orf35
1	C1orf198
1	C1orf123

1 C1orf106
1 C19orf70
1 C19orf47
1 C19orf35
1 C19orf22
1 C18orf21
1 C18orf19
1 C17orf85
1 C17orf76
1 C17orf39
1 C17orf28
1 C15orf55
1 C15orf44
1 C15orf42
1 C15orf29
1 C15orf27
1 C15orf23
1 C14orf37
1 C14orf101
1 C12orf66
1 C12orf41
1 C12orf34
1 C12orf26
1 C12orf24
1 C11orf95
1 C11orf2
1 C10orf47
1 C10orf18
1 C10orf107
1 BUD13
1 BUB3
1 BTN3A1
1 BTBD8
1 BTBD10
1 BTAF1
1 BSPRY
1 BSG
1 BRSK1
1 BRPF3
1 BRD8
1 BRD4
1 BRCA2
1 BPTF
1 BPIFC
1 BORA
1 BOLA1
1 BNIP2
1 BMPER
1 BLMH
1 BICD2
1 BICD1
1 BICC1
1 BHMT2

1 BECN1
1 BDH1
1 BCL9
1 BCL2L2
1 BCL11A
1 BCAS2
1 BCAN
1 BCAM
1 BBS7
1 BBS2
1 BBC3
1 BAZ2B
1 BAZ1B
1 BARHL2
1 BARD1
1 BAIAP3
1 BAG4
1 B4GALNT3
1 B3GNT1
1 B3GALT6
1 B3GALT2
1 AZIN1
1 AXL
1 AXIN2
1 AXIN1
1 AVPR1A
1 AVP
1 AVIL
1 AURKAIP1
1 ATXN7L3
1 ATXN2L
1 ATXN1L
1 ATRNL1
1 ATR
1 ATP8B4
1 ATP8A2
1 ATP6V1B2
1 ATP6V1A
1 ATP6V0D2
1 ATP5O
1 ATP5J2
1 ATP5D
1 ATP5B
1 ATP2B2
1 ATP2B1
1 ATP1A4
1 ATP13A5
1 ATP13A3
1 ATOH7
1 ATN1
1 ATM
1 ATL2
1 ATL1

1 ATF2
1 ATAD2
1 ASRGL1
1 ASNSD1
1 ASL
1 ASCL2
1 ASCC2
1 ASB5
1 ASB4
1 ASB3
1 ASB15
1 ASAP3
1 ARSA
1 ARRB1
1 ARPC5L
1 ARPC4
1 ARPC1A
1 ARNTL
1 ARMC5
1 ARMC2
1 ARMC1
1 ARL6
1 ARL2BP
1 ARL14
1 ARHGEF9
1 ARHGEF7
1 ARHGEF5
1 ARHGEF40
1 ARHGEF4
1 ARHGEF26
1 ARHGEF2
1 ARHGEF11
1 ARHGAP9
1 ARHGAP6
1 ARHGAP35
1 ARHGAP29
1 ARHGAP24
1 ARHGAP17
1 ARHGAP11A
1 ARHGAP10
1 ARGLU1
1 ARFIP2
1 ARFGEF2
1 ARFGAP2
1 APTX
1 APPL2
1 APOOL
1 APOB
1 APOA5
1 APLP1
1 APBA3
1 APBA1
1 APAF1

1 AP4E1
1 AP3B2
1 AP2B1
1 AP1M1
1 AP1G1
1 ANXA4
1 ANXA10
1 ANXA1
1 ANTXR1
1 ANP32B
1 ANO5
1 ANO4
1 ANO1
1 ANKS3
1 ANKRD50
1 ANKRD32
1 ANKRD29
1 ANKRD2
1 ANKRD17
1 ANKRD12
1 ANKRD11
1 ANKMY2
1 ANKLE2
1 ANKFN1
1 ANGPTL3
1 ANGEL2
1 ANGEL1
1 ANAPC5
1 ANAPC4
1 ALPK1
1 ALOX15B
1 ALKBH8
1 ALDH16A1
1 ALB
1 AKR1E2
1 AKAP6
1 AKAP13
1 AKAP12
1 AK8
1 AK2
1 AIFM1
1 AIF1
1 AHSG
1 AGRN
1 AGPHD1
1 AGPAT5
1 AGPAT2
1 AGMAT
1 AGK
1 AGBL2
1 AGAP2
1 AGAP1
1 AFTPH

1 AFP
1 AFF1
1 AES
1 AEBP1
1 ADRBK2
1 ADPRHL2
1 ADK
1 ADI1
1 ADCYAP1R1
1 ADCY5
1 ADCY1
1 ADCK4
1 ADARB2
1 ADAR
1 ADAMTSL3
1 ADAMTS2
1 ADAMTS17
1 ADAMTS16
1 ADAMTS15
1 ADAMTS10
1 ADAM9
1 ADAM17
1 ADAM12
1 ADAL
1 ADAD2
1 ACY1
1 ACTR10
1 ACSS3
1 ACSL5
1 ACSL1
1 ACPT
1 ACPP
1 ACOT4
1 ACOT12
1 ACO2
1 ACO1
1 ACMSD
1 ACE2
1 ACCN5
1 ACCN4
1 ACCN1
1 ACBD3
1 ACAP3
1 ACAP1
1 ACADSB
1 ACADM
1 ACAD9
1 ACAD8
1 ACACA
1 ABR
1 ABLIM3
1 ABI2
1 ABHD8

1	ABHD13
1	ABCF3
1	ABCB6
1	ABCB4
1	ABCB11
1	ABCB1
1	ABCA7
1	ABCA5
1	ABCA4
1	ABCA2
1	ABCA13
1	AAK1
1	A2ML1
1	A2M
1	A1CF
