# Supplementary material for: SIMPLEX: Cloud-Enabled Pipeline for the Comprehensive Analysis of Exome Sequencing Data
Source: PLoS One. 2012 Aug 1;7(8):e41948. doi: 10.1371/journal.pone.0041948 (PMC3411592; doi:10.1371/journal.pone.0041948)
Supplement: Supplementary Material S1 — SIMPLEX user manual. (PDF) [file pone.0041948.s007.pdf]

SIMPLEX
User Manual

Version 1.0
May 3, 2012

Andreas Dander
Maria Fischer
Stephan Pabinger
Rene Snajder
Gernot Stocker

Innsbruck Medical University
Division for Bioinformatics
Innrain 80
6020 Innsbruck, Austria
http://www.icbi.at

Contents

1. Introduction **4**
1.1. SIMPLEX 4

2. Download and installation **5**
2.1. SIMPLEX Server 5
2.1.1. Virtualization image (Virtual machine image) 5
2.1.2. Cloud image 5
2.1.3. Passwords 6
2.2. SIMPLEX Client 6
2.2.1. Java 6 Support 6

3. EC2 Cloud Usage **7**
3.1. Prerequisites 7
3.2. Preparations 7
3.3. Starting the SIMPLEX AMI 8
3.4. Initializing the SIMPLEX Cluster 9

4. Usage **10**
4.1. Input files 10
4.1.1. Illumina reads 10
4.1.2. SOLiD reads 10
4.1.3. Naming convention for sequence read files 10
4.1.4. Exome definition 11
4.1.5. Server connection properties file 11
4.2. Calling SIMPLEX 12
4.2.1. Illumina Single End 12
4.2.2. Illumina Single End with prepocessing and quality report 13
4.2.3. Illumina Paired End 13
4.2.4. SOLiD Single End 13
4.2.5. SOLiD Paired End 14
4.2.6. Results 15
4.2.7. Output 16

5. Software development **18**
5.1. Clusterservice definition 18
5.2. Clusterresource definition 20
5.3. Java classes 21
5.3.1. CSCommand and PipelineJobFactory 21
5.3.2. Splicing 24

5.3.3. Pipeline 26
5.4. Integrate new species 27
5.4.1. Server side adaptations 27
5.4.2. Adaptations to the source code 29

A. FAQ **30**
A.1. Change password 30
A.2. What can I do if simplex prints “Unable to connect to JClusterService.” 30
A.3. ToDo 30

1. Introduction

This document is the user manual describing the automatic analysis pipeline for exome sequencing data called SIMPLEX. The pipeline can be downloaded from http://simplex.i-med.ac.at and is distributed under the GPL.

1.1. SIMPLEX

SIMPLEX is an autonomous analysis pipeline, which comprises the complete exome sequencing analysis workflow, including the following steps: (1) initial quality control, intelligent data filtering and pre-processing, (2) sequence alignment to a reference genome and refinement, (3) alignment statistics; (4) SNP and DIP detection, (5) functional annotation of variants using different approaches, and report generation during various stages of the workflow (see figure 1.1).

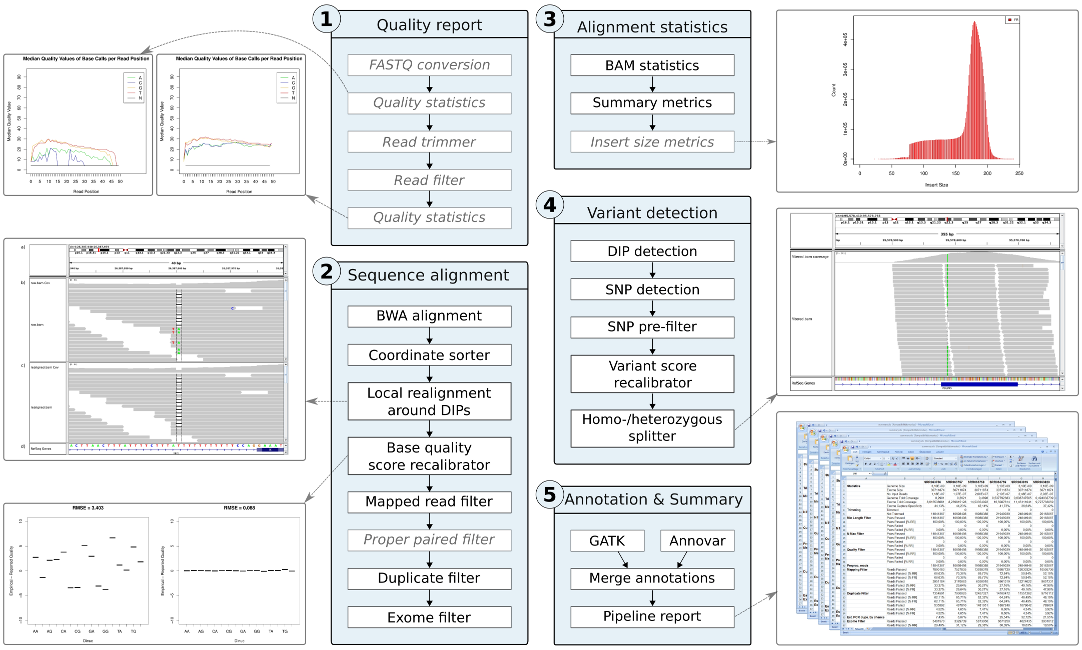

Figure 1.1.: **SIMPLEX analysis pipeline overview:** Mandatory components are depicted in black, optional in gray.

SIMPLEX connects the selected analysis steps, exposes all available parameters for customized usage, performs required data handling, and distributes computationally expensive tasks either on a dedicated high-performance computing infrastructure or on the Amazon cloud environment (EC2).

2. Download and installation

2.1. SIMPLEX Server

To run SIMPLEX you need both an instance of the server, which performs the analysis, and the client, which transfers data from and to the server and handles the flow of the pipeline. Since setting up the SIMPLEX server would be time consuming and complicated, we provide both a fully configured virtual machine image (in OVA format) as well as a Amazon EC2 cloud image.

2.1.1. Virtualization image (Virtual machine image)

The following steps should be performed to get a running instance of SIMPLEX.

1. Download the image at http://simplex.i-med.ac.at
2. Start your virtualization software (every software supporting Open Virtualization Format should work)[1]
3. Load the downloaded image into the virtualization software
4. Start the image
5. Configure networking between host and guest system using user:
   `Username:vagrant; Password:vagrant`
6. You can get your ip-adress using command `ifconfig` (e.g. 10.0.0.2)
7. Open a browser with https://your_ip/JClusterService. If you get a confusing xml page you're done!

2.1.2. Cloud image

The Cloud image of SIMPLEX is based on the CloudMan image of Galaxy[2]. It can be instantiated from the Amazon AWS Console. The AMI name is "Simplex 1.02 Ubuntu 10.04 LTS based on CBL".

Detailed instructions on how to use SIMPLEX within the cloud are given in section 3.

[1] We tested it on Oracle VM VirtualBox version 4.1.8 http://www.virtualbox.org

[2] For more information please visit http://wiki.g2.bx.psu.edu/Admin/Cloud.

2.1.3. Passwords

As explained in section 4.1.5 you need a username & password to access the SIMPLEX cluster (Whether it is the VirtualBox image or the cloud image..).

For both, the VirtualBox and the cloud image, the following default is used:

```
Username: simplex
Password: simplexdemo
```

2.2. SIMPLEX Client

The SIMPLEX Client can run on any office computer. Please perform the following steps to install SIMPLEX:

1. Download the SIMPLEX Client at http://simplex.i-med.ac.at
2. Install Oracle Java Runtime 7 (recommended) or 6 from http://www.oracle.com
3. See section 4 on how to use SIMPLEX
4. See subsection 4.1.5 on how to gernerate the properties file, which specifies the connection to the server

2.2.1. Java 6 Support

SIMPLEX works best with Java 7 installed. If you want to use it on Java 6, you need to perform a few more steps:

1. Download the Java 6 support libraries for Simplex from http://simplex.i-med.ac.at
2. Create a directory in your JAVA_HOME directory with the following path: JAVA_HOME/jre/lib/endorsed/
3. Unpack the tgz file into the directory you just created
4. When calling Simplex, make sure you use the same Java version you just extended.

3. EC2 Cloud Usage

We created an AMI (Amazon Machine Image) containing SIMPLEX. This AMI is based on CloudBioLinux[1] and therefore uses CloudMan [1] for resource management.

3.1. Prerequisites

The only thing you need to start SIMPLEX in the Cloud is an EC2 Account. You can get one at http://aws.amazon.com/console/. Basic understanding of how to use the AWS Management Console is recommended. But as we will be giving a step-by-step instruction on how to use it, it is not entirely necessary. For easy registration without waiting times a credit card is required. Otherwise, if you intend to use it more often, you can also contact a sales representative and ask for them to send you a monthly bill instead.

3.2. Preparations

You will only have to perform these steps if you are using SIMPLEX for the first time. If you have used the CloudBioLinux or Galaxy in the Cloud before, you'll already have all required settings. Therefore, you could skip this step. What you have to do is to create a security group for our Instances and assign the proper rules. The following steps are the same as described in "Step 1" in the CloudMan Manual [2] with the addition of one rule (HTTPS):

- Create an EC2 account and log-in to the AWS management Console
- In the EC2 Tab, click "Security Groups" (on the left hand side in "Network and Security").
- Click "Create Security Group".
- Enter a name and description (for example "SIMPLEX").
- After the Group has been created, select the Group in the list and click the "Inbound" tab in the lower panel.
- Create new rules using the following values in the fields on the left side (Create a new rule, Port range, Source) and click "Add Rule":

[1] http://cloudbiolinux.org/

[2] http://wiki.g2.bx.psu.edu/Admin/Cloud#Detailed_steps

  - Type: HTTP; Source: 0.0.0.0/0 (or you can enter your public IP address here if you want to limit access)
  - Type: HTTPS; Source: 0.0.0.0/0 (or you can enter your public IP address here if you want to limit access)
  - Type: SSH; Source: 0.0.0.0/0 (or you can enter your public IP address here if you want to limit access)
  - Type: Custom TCP Rule; Port range: 42284; Source: 0.0.0.0/0
  - Type: Custom TCP Rule; Port range: 20-21; Source: 0.0.0.0/0
  - Type: Custom TCP Rule; Port range: 30000-30100; Source: 0.0.0.0/0
  - Type: All TCP; Source: the name of the security group, e.g. "SIMPLEX"
- Click "Apply Rule Changes"

3.3. Starting the SIMPLEX AMI

- Login to the AWS Management Console and click the EC2 tab.
- Click "AMIs" in the Navigation panel on the left side
- Search for "SIMPLEX" in "All Images". If there is more than one result, please choose the one from Owner "372123314130", which is us.
- Click "Launch" to start the instance.
  - Select Instance Type "Extra Large" (8 ECUs) or higher (But NOT High-Memory Extra Large). Basically, the instance you start must have at least 4 CPU cores, otherwise some jobs will be queued forever. Click Continue.
  - On the next page, in the "User Data" field you must provide the following information:

```
cluster_name: <a name for the SIMPLEX cluster>
password: <a password for the CloudMan webinterface>
access_key: <your AWS access key>
secret_key: <your AWS secret key>
```

    You got the AWS access key and AWS secret key when you created your Amazon Account. You can also retrieve it by clicking your username in the upper right corner and then "Security credentials" (must be logged in to a power user. For example it might look like this:

```
cluster_name: mysimplexcluster
password: supersecurepw
access_key: AEIQEJ5RUZDHNWS69IJZ
secret_key: DsopjdJSOdjqShkqhkw+DJQJKL+JD781Auo8D7Aj
```

- Click Continue. On the next page you can select an existing key pair or create a new one. This step is only required if you plan to log on to the instance via SSH.
- Click Continue. Now you are in the "Configure Firewall" page. Select the Security Group you configured in the Preparations chapter.
- Click Continue and then Launch. Your instance should be launching.

3.4. Initializing the SIMPLEX Cluster

Now that the instance is starting, we have to open the CloudMan interface to initialize the Cluster in the Cloud.

- Click "Instances" on the left side, and select the instance from the list. In the bottom panel the parameters of this instance are displayed.
- See the "Public DNS:" field. It will show something like "ec2-50-19-171-174.compute-1.amazonaws.com". This is the public domain name of your instance.
- Open a new browser window, and enter the public domain name of your instance in the address bar. For example: http://ec2-50-19-171-174.compute-1.amazonaws.com
- If you cannot connect, try to give it a few minutes to start up. If after about 2 or 3 minutes you still cannot connect, check if the Security Group settings are set properly.
- You should now be asked for a username and password. The user name is "CM Administration" and the password is the one you set in the "User Data" field (in our example it was "supersecurepw".
- Now you will see the "Initial Cluster Configuration" dialog of CloudMan. Enter the number of GB you want to reserve on the data volume. This is important, as it depends on how big the data is you want to analyze with SIMPLEX. If you have 1GB input data, reserve at least 10GB in the Cloud as all the in-between results will also be stored there.
- Click "Start Cluster". Now the CloudMan and Galaxy services are starting up. Wait until both "Application" and "Data" have a green dot displayed (you may want to refresh the page from time to time). This may take a couple of minutes.
- Once the cluster initialization is done, you should be able to access https://–public instance domain name–/JClusterService. It should show an XML file. Don't bother trying to understand it. If it's there, initialization is completed.

Congratulations! The SIMPLEX cluster is running in your very own cloud instance!

You can now use this URL to in the cluster.properties file. See Section 4 on how to use Simplex.

4. Usage

SIMPLEX is designed as an intuitive, easy to use, and highly customizable command-line program to provide analyses for single end (SE) and paired end (PE) as well as Illumina (NS) and SOLiD (CS) experiments. All available parameters are listed in a document which can be downloaded at http://www.icbi.at/exome.

4.1. Input files

To analyze exome sequencing data with SIMPLEX, sequence read files, the exome (or target region) definition file, and server connection properties are needed. An example how to pass these files is given in section 4.2.

4.1.1. Illumina reads

Illumina raw reads (provided by parameter **-I**) are expected to be in FASTQ format. All three types, Sanger, Solexa (aka. Illumina 1.3-), and Illumina 1.3+ FASTQ, are supported by the pipeline. A detailed description of the differences between these format types is given in [2] and on http://en.wikipedia.org/wiki/FASTQ_format.

CAVEAT: to avoid interferance with downstream analyses, enable FASTQ conversion (parameters **-fqc** and **-cf** [**solexa** | **illumina**]) if Solexa or Illumina FASTQ format is used. This will trigger the conversion into Sanger FASTQ format before any other analysis steps are performed.

4.1.2. SOLiD reads

SOLiD data is provided to the pipeline by two (SE) or four (PE) files: csfasta for sequence information (parameter **-I**) and qual files (parameter **-Q**) containing each read's corresponding quality scores.

4.1.3. Naming convention for sequence read files

In order to generate reasonable output file names, SIMPLEX requires read input files to be named according to the following patterns:

NS/SE

runname.fq[.gz] - read data in FASTQ format

NS/PE

*runname*_R1.fq[.gz] - first reads of pairs in FASTQ format

*runname*_R2.fq[.gz] - second reads of pairs in FASTQ format

CS/SE

runname.csfasta[.gz] - read sequences in FASTA format

*runname*_QV.qual[.gz] - read quality values in FASTA format

CS/PE

*runname*_F3.csfasta[.gz] - sequence of first reads of pairs in FASTA format

*runname*_F3_QV.qual[.gz] - quality values of first reads of pairs in FASTA format

*runname*_F5.csfasta[.gz] - sequence of second reads of pairs in FASTA format

*runname*_F5_QV.qual[.gz] - quality values of second reads of pairs in FASTA format

4.1.4. Exome definition

This file (provided by parameter **-sfeb**) is needed to determin fold coverage measurments (e.g. overall fold coverage, identification of not/low captured regions) and to filter non-exonic/not targetted reads. It defines all exons/target regions by their positions within the genome (chromosome, start, and end position) in BED file format[1]. Idealy, the exon's name should include further description about corresponding gene name, exon number, and ccds id, e.g.:

```
track name=CCDS description="chrom start end name score strand"
chr1 69090 70008 OR4F5;Exon1;CCDS30547.1 850 +
chr1 367658 368597 OR4F29;Exon1;CCDS41220.1 850 +
chr1 621095 622034 OR4F16;Exon1;CCDS41221.1 850 -
chr1 861321 861393 SAMD11;Exon1;CCDS2.2 850 +
```

We do provide an exome definition files based on the released CCDS update for human build HsGRCh37.3 (September 7, 2011) which can be downloaded from the SIMPLEX webpage http://simplex.i-med.ac.at.

4.1.5. Server connection properties file

The server connection properties file (provided by parameter **-k** or **–clusterpropsfile**) is required to tell SIMPLEX where to contact the server instance. This could be either the VirtualBox image, or the AMI Cloud Image or - if you chose to set one up by yourself - your own server running JClusterService.

The format of the file looks like this:

```
url=https://URL_TO_YOUR_CLUSTER/JClusterService
user=username
password=password
```

[1] http://genome.ucsc.edu/FAQ/FAQformat.html#format1

For example: if you run the SIMPLEX Amazon Cloud Image, it would look like this (replace "ec2-50-19-171-174.compute-1.amazonaws.com" with your instance's public DNS):

```
url=https://ec2-50-19-171-174.compute-1.amazonaws.com/JClusterService
user=simplex
password=simplexdemo
```

4.2. Calling SIMPLEX

Depending on library preparation and sequencing technology used, different minimum parameter sets are required to start the pipeline. The following examples describe a minimal **testdata** pipeline call to test SIMPLEX with the provided testdata available on http://simplex.i-med.ac.at. We suggest to put the actual pipeline call into a script as it simplifies reproducing results.

As the following examples should run fast, a subset of the genome is integrated into SIMPLEX. This subset of hg19 (`hg19.test`) contains just the human chromosomes 1 and 21. To specify this small genome the user can start SIMPLEX using `hg19.test` as Illumina reference `hg19.color.test` as SOLiD reference and `CCDS.20110907.test.bed` as the exome specification, for a real run use `hg19`, `hg19.color` and `CCDS.20110907.bed` respectively.

4.2.1. Illumina Single End

```
java -jar simplex.jar -c exomeSE -genP hg19.test -I testdata_R1.fq.gz \
   -od path_to_output_directory -sfeb CCDS.20110907.test.bed -fqc\
   -dsP 80 -k connection.properties
```

Parameter description:

-c exomeSE ... use SE pipeline

-genP hg19.test ... prefix of the test reference genome

-I testdata_R1.fq.gz ... input file in FASTQ format

-od path_to_outputdirectory ... output directory

-sfeb CCDS.20110907.test.bed ... bed file specifying the test exome

-fqc ... conversion to standard SANGER fastq

-dsP 80 ... percentage to distinguish between homo- and heterozygous DIPs

-k connection.properties ... server connection properties of the service

4.2.2. Illumina Single End with prepocessing and quality report

```
java -jar simplex.jar -c exomeSE -genP hg19.test -I testdata_R1.fq.gz \
   -od path_to_output_directory -sfeb CCDS.20110907.test.bed -fqc\
   -dsP 80 -k connection.properties \
   -N 0.05 -m 25 -sc raw_report,filter_report
```

Additional parameter description:

-N 0.05 ... remove reads with more then 5% Ns

-m 25 ... remove reads shorter then 25

-sc raw_report,filter_report ... quality report for raw and preprocessed data

4.2.3. Illumina Paired End

```
java -jar simplex.jar -c exomePE -I testdata_R1.fq.gz,testdata_R2.fq.gz \
   -od path_to_output_directory -genP hg19.test -fqc\
   -sfeb CCDS.20110907.test.bed -dsP 80 -k connection.properties
```

Parameter description:

-c exomePE ... use PE pipeline

-genP hg19.test ... prefix of the test reference genome

-I testdata_R1.fq.gz,testdata_R2.fq.gz ... first and second read in pair input files in FASTQ format

-od path_to_outputdirectory ... output directory

-fqc ... conversion to standard SANGER fastq

-sfeb CCDS.20110907.test.bed ... bed file specifying the test exome

-dsP 80 ... percentage to distinguish between homo- and heterozygous DIPs

-k connection.properties ... server connection properties of the service

4.2.4. SOLiD Single End

```
java -jar simplex.jar -c exomeSE -CS -genP hg19.color.test \
   -I testset_F3.csfasta -Q testset_F3_QV.qual \
   -od path_to_output_directory -sfeb CCDS.20110907.test.bed \
   -dsP 80 -k connection.properties
```

Parameter description:

-c exomeSE ... use SE pipeline

-*CS* ... analyse color space data

-*genP hg19.color.test* ... prefix of the test reference genome

-*I testset_F3.csfasta.gz* ... SOLiD read sequences in FASTA format

-*Q testset_F3_QV.qual.gz* ... SOLiD read quality values in FASTA format

-*od path_to_outputdirectory* ... output directory

-*sfeb CCDS.20110907.test.bed* ... bed file specifying the test exome

-*dsP 80* ... percentage to distinguish between homo- and heterozygous DIPs

-*k connection.properties* ... server connection properties of the service

4.2.5. SOLiD Paired End

```
java -jar simplex.jar -c exomePE -CS -od path_to_output_directory\
   -I pe_testset_F3.csfasta,pe_testset_F5.csfasta \
   -Q pe_testset_F3_QV.qual,pe_testset_F5_QV.qual \
   -genP hg19.color.test -sfeb CCDS.20110907.test.bed \
   -dsP 80 -k connection.properties
```

Parameter description:

-*c exomePE* ... use PE pipeline

-*CS* ... analyse color space data

-*genP hg19.color.test* ... prefix of the test reference genome

-*I pe_testset_F3.csfasta,pe_testset_F5.csfasta* ... first and second read in pair SOLiD read sequences in FASTA format

-*Q pe_testset_F3_QV.qual,pe_testset_F5_QV.qual* ... first and second read in pair SOLiD read quality values in FASTA format

-*od path_to_outputdirectory* ... output directory

-*sfeb CCDS.20110907.test.bed* ... bed file specifying the test exome

-*dsP 80* ... percentage to distinguish between homo- and heterozygous DIPs

-*k connection.properties* ... server connection properties of the service

4.2.6. Results

These minimal parameter sets would cause SIMPLEX to

- calculate raw coverage statistics,
- align the given reads to a test subset of hg19 (chr1 and chr21, defined as SIMPLEX's test reference genome),
- local realign the reads around indels,
- recalibrate quality scores
- filter unmapped reads, duplicates, and not on target reads,
- calculate alignment metrics,
- identify and annotate DIPs (in vcf and tab delimited files),
- identify and annotate SNPs (in vcf and tab delimited files),
- generate indexes for vcf files to be viewed in genome browsers, and
- generate a detailed summary report (available as tab delimited and xls file)

Additional parameter settings (e.g. filtering parameters) are available to enable preprocessing of raw reads, including

- FASTQ conversion
- raw and filtered read statistics including
  - quality value metrics
  - nucleotide distribution
  - N content
- raw read trimming (currently available for Illumina reads only)
- raw read filtering based on (currently available for Illumina reads only)
  - N content
  - read length
  - quality values

4.2.7. Output

The following list describes the generated output files during a testrun with Illumina SE data (section 4.2.2). Note that the output varies depending on the given input files and selected parameters. The steps are explained in the log file generated in the result directory.

- **Step0** - Conversion of fastq files to standard SANGER fastq
- **Step1** - Preparation step for quality report
- **Step2** - Quality report on **raw** sequence reads - output is in [pdf] format
- **Step3** - Result files of filtering (min-length, n-max, quality, ...) and the generated [log] files
- **Step4** - Statistics: the produced file contains basic information about the input files (genome-size, number of sequences, number of total bases) [txt]
- **Step5** - Preparation step for quality report
- **Step6** - Quality report on **filtered** sequence reads - output is in [pdf] format
- **Step7** - Output of the BWA alignment for each input file - generates [sai] and [log] files
- **Step8** - Component of BWA to generate alignments in the SAM format - outputs a [log] file
- **Step9** - Sorted alignment [bam] file - can be readily loaded into genome viewers
- **Step10** - Output of the local realignment step around indels - Sorted, realigned [bam] file - can be readily loaded into genome viewers; [log] files produced during the realignment process
- **Step11** - Quality score recalibrated [bam] file - output is also present in [csv] format
- **Step12** - Contains [bam] file with only reads marked as mapped. Furthermore, all filtered reads are stored in an additional [bam] file.
- **Step13** - Result of duplicate filtering - file is in [bam] format. Moreover, [log] file for this step is generated.
- **Step14** - Generated [bam] file contains only reads that were mapped on the specified target region. This file is then used for variant calling and is therefore very well suited for graphical display in genome viewers. The generated [log] file contains useful information about capture specificity and coverage.
- **Step15** - Basic statistics about the generated bam file
- **Step16** - Alignment statistics - [metrics] file

- **Step17** - *No output produced*
- **Step18** - Exone coverage statistics file [log] - based on the generated [exonecov] file
- **Step19** - Output of DIP calling - [vcf], [bed] files with identified variants
- **Step20** - Only [log] files produced
- **Step21** - Annovar and GATK annotated DIP vcf files
- **Step22** - Homo-/heterozygous DIPs in [txt] and [vcf] format
- **Step23** - Output of SNP calling - [vcf] files with identified variants
- **Step24** - Filtered SNP files in [vcf] format
- **Step25** - Only [log] files produced
- **Step26** - Annovar and GATK annotated DIP vcf files
- **Step27** - Recalibrated SNP vcf files
- **Step28** - Homo-/heterozygous SNPs in [txt] and [vcf] format
- **Step29** - This steps contains the final variant files. DIP and SNP files are available in [vcf] format or in the tab delimited [amt] format, which can be easily loaded into a spreadsheet program such as Excel. Moreover, for all vcf files the corresponding indices are generated.
- **Step30** - Contains [log] files about the vcf indices that are produced in all previous steps.
- **Step31** - This step contains the pipeline summary file in txt or Excel format.

Additional steps

- **StepX** - Result files of trimming (length, N) and the generated [log] files

5. Software development

In order to integrate additional command-line analysis tools into SIMPLEX the following steps are required:

1. Install the selected command-line tool on the server.
2. Checkout SIMPLEX source code (subversion links are provided at http://simplex.i-med.ac.at).
3. Extend *clusterservice_definition.xml* to reflect the tool's parameter settings, input, and output files (see section 5.1).
4. If needed, define path to additionally downloaded resource files/databases (e.g. bwa index) on the server in *clusterresource-definition.properties*. This will allow you to link to the resources in *clusterservice_definition.xml* (see section 5.2).
5. Create Java class *ToolNameCSCommand* which checks and sets all parameters and transfers input/output files to/from the server.
6. Update method *getInstance(int, Object)* in Java class *PipelineJobFactory* to return an instance of *ToolNameCSCommand*.
7. Write Java class *ToolNameSplicing* which acts as a wrapper between the pipeline's and the tool's parameters.
8. Integrate tool in *ExomeBwaSEPipeline* and/or *ExomeBwaPEPipeline* which are the Java pipeline classes for SE and PE data, respectively.

5.1. Clusterservice definition

The XML file *clusterservice_definition.xml* acts as a link between command line tools installed on the server and SIMPLEX Java classes. It represents the available applications including their parameters, input-, and outputfiles. In order to add another application, an XML clusterservice element needs to be specified which contains the following attributes and child elements:

- clusterservice attributes
  - name - program name used by Java classes (e.g. *PipelineJobFactory*) to reference the command line tool
  - program-path - path to program on server
  - nodes - defines how many nodes are booked by the queueing system to execute the program call

    – validate - check parameters

- clusterservice elements
    – description - simple description
    – parameterlist - list of used/set parameter child elements with the following attributes
        * name - parameter name used in Java CSCommand class
        * switch - defines switch (parameter) used in command line
        * position - defines at which position the switch should be placed
        * mandatory - true for required parameters
    – inputfilelist - list of inputfiles transferred to the server
    – resultfilelist - list of resultfiles which can be downloaded by the client

The following XML snippet demonstrates how a clusterservice element is defined in SIMPLEX (in this case the program 'bwa aln' is described which is called by *bwa aln [options] ⟨prefix⟩ ⟨in.fq⟩*).

clusterservice_definition.xml: example clusterservice element

```
<clusterservice name=”BWA_ALN” program−path=”/usr/local/bioinf/bwa/bwa−0.5.10/bwa aln” nodes=”5” validate=”false”>
  <description>bwa aln v0.5.10</description>
  <parameterlist>
    <parameter name=”bwaan” switch=”−n” position=”0” mandatory=”false” />
    <parameter name=”bwaao” switch=”−o” position=”1” mandatory=”false” />
    <!−− use 5 nodes for computation −−>
    <parameter name=”bwaat” switch=”−t” position=”2” mandatory=”true”>
      <parameter−value isdefault=”true”>5</parameter−value>
    </parameter>
    ...
    <!−− resulting sai index file −−>
    <parameter name=”bwaaR” switch=”−f” position=”21” mandatory=”true”>
      <parameter−value type=”resultfile” name=”saifile” isdefault=”true” />
    </parameter>
    <!−− link to prefix −−>
    <parameter name=”bwaaP” position=”22” mandatory=”true” resource−namespace=”bwa_reference” />
    <!−− input file −−>
    <parameter name=”bwaaI” position=”23” mandatory=”true”>
      <parameter−value name=”bwaaI” type=”inputfile” isdefault=”true” />
      <parameter−value name=”outputfile” type=”resultfile” externalservice=”FASTQ_CONVERTER” />
    </parameter>
  </parameterlist>
  < inputfilelist >
    <inputfile name=”bwaaI”>##−STD_INPUTFILE−##.fq</inputfile>
  </ inputfilelist >
```

```
26   < resultfilelist >
27     < resultfile  name="stdout">##-STD_OUTPUTFILE-##</resultfile>
28     < resultfile  name="stderr">##-STD_ERRORFILE-##</resultfile>
29     < resultfile  name="saifile">##-RESULTFILE-##</resultfile>
30   </ resultfilelist >
31 </clusterservice>
```

Line 1 - defines clusterservice 'BWA_ALN' (*name*) which is called on the server by '/usr/local/bioinf/bwa/bwa-0.5.10/bwa aln' (*program-path*) and books '5' *nodes* for its execution.

Line 3-22 - list of parameters which can be referred by Java classes (see section 5.3)

Line 4 - optional bwa aln parameter '-n' (*switch*) which, if given, will be placed immediately after the command '/usr/local/bioinf/bwa/bwa-0.5.10/bwa aln' (*position="0"*) and can be referred in Java classes by 'bwaan' (*name*).

Line 7 - required parameter '-t' with its given default value '5' (element *parameter-value* in combination with attribute *isdefault="true"*).

Line 12-21 - file parameters (reference index, input -, and output files) passed to bwa aln

Line 12-14 - result file parameter (*type="resultfile"*) 'saifile' (*name*) defined in the *resultfilelist* element.

Line 16 - required parameter 'bwaaP' (*name*) accepting values representing a resource file stored on the server (*resource-namespace*). Resource-namespace values must be defined in *clusterresource-definition.properties* specifying the namespace (= name used by clusterservice definition), the namespace's possible values and their corresponding file paths on the server.

Line 18 - 21 - required parameter 'bwaaI' which either uses an uploaded inputfile or a file which is the result of a previously executed program called 'FASTQ_CONVERTER' (*externalservice*). 'FASTQ_CONVERTER' must be specified in the clusterservice definition as well.

Line 23 - 25 - list of files which can be uploaded to the server.

Line 26 - 30 - list of resultfiles on the server. ##-STD_INPUTFILE-##, ##-STD_OUTPUTFILE-##, and ##-STD_ERRORTFILE-## are used as placeholders for stdin, stdout, and stderr on the server.

5.2. Clusterresource definition

Cluster resources describe available databases/files on the server. In contrast to ordinary input files, these resources are used regularly by more than one program call (e.g. genome reference, genome reference index for alignment programs, dbSNP for annotation, etc.)

and therefore should be stored permanently on the server. A cluster resource is defined by its namespace, available values, and its absolute file path. In the example below 'bwa_reference' is the namespace which can be used by clusterservice definitions to declare resource parameters and 'hg18' and 'hg19' are the parameter's possible values.

Listing 5.1: clusterresource-definition.properties

```
...
# BWA
bwa_reference:hg18 =/export/data/genomes/hg18/programs/bwa/0.5.10/ucsc.pa.ns.fa
bwa_reference:hg19=/export/data/genomes/hg19/programs/bwa/0.5.10/ucsc.pa.ns.fa
...
```

5.3. Java classes

5.3.1. CSCommand and PipelineJobFactory

A cluster service command (CSCommand) Java class needs to be implemented to represent a command line tool and to handle all parameter, input, and output settings on the client side. The class must extend *AbstractClusterPipelineCommand* and implement the methods

getCommandName - command name used in clusterservice_definition.xml

checkParameters - are parameter values valid?

initializeCommandImplementation - sets parameters, registers files for up- and download

The following examples shows how a CSCommand class should be implemented and how it should be integrated in the PipelineJobFactory.

Listing 5.2: CSCommand example

```
/** Project: CommonBioCommands */
...
/**
 * Bwa version: 0.5.10
 * CSCommand class for bwa (Burrow Wheeler Alignment) subprogram aln
 */
public class BwaAlnCSCommand extends AbstractClusterPipelineCommand {

  public final static String CMD_NAME = "BWA_ALN";
  @Override
  public String getCommandName() {
    return CMD_NAME;
  }
  // parameter definitions
  @CommandOption(longOption = "bwaa-max-ed", isSwitch = false, description = "
      Maximum edit distance. [default: 0.04]")
```

```
public final static String OPT_MED = "bwaan"; // same parameter name as in clusterservice_definition.xml

@CommandOption(longOption = "bwaa-max-go", isSwitch = false, description = "Maximum number of gap opens. [default: 1]")
public final static String OPT_MGO = "bwaao";
...
@CommandOption(longOption = "bwaa-inputfile", required = true, isSwitch = false, description = "Sequence read file in Fastq format.")
public final static String OPT_INPUT = "bwaaI";

@CommandOption(longOption = "bwaa-prefix", required=true, isSwitch = false, description = GenomeReferenceHelper.OPTION_MSG)
public final static String OPT_GENOME = "bwaaP";

@CommandOption(longOption = "bwaa-result", required = true, isSwitch = false, description = "Prefix of the resultfile.")
public final static String OPT_RESULT = "bwaaR";

// returns error string if a parameter is not set correctly, otherwise null
@Override
public String checkParameters(CommandLine command) {
  String error = checkFileParameterValidity(command, OPT_INPUT);
  if (error != null) {
    return error;
  }
  ...
  error = checkOptDoubleParameterValidity(command, OPT_MED);
  if (error != null) {
    return error;
  }

  error = checkOptIntParameterValidity(command, OPT_MGO);
  if (error != null) {
    return error;
  }
  ...
  return null;
}

@Override
protected String initializeCommandImplementation(CommandLine command, Options parameters) {
  // create job
  ClusterJobInterface bwaJob = getJobFactory().getInstance(PipelineJobFactory.BWA_ALN);
  Map<String,ParameterValueInterface> parameterMap = initParameterMap(bwaJob);

  // set parameters and input files
```

```
    try {
      setInputFileByName(command, parameterMap, bwaJob, OPT_INPUT);
      // add clusterservice resource
      if (command.hasOption(OPT_GENOME)) addResourceValueToParameterMap(
          command, parameterMap, OPT_GENOME);
      // add ordinary parameters if given
      if (command.hasOption(OPT_MED)) addToParameterMap(command, parameterMap
          , OPT_MED);
      if (command.hasOption(OPT_MGO)) addToParameterMap(command, parameterMap
          , OPT_MGO);
      ...
      bwaJob.setParameters(parameterMap);
    } catch (IOException e) {
      return e.getMessage();
    } catch (ClusterJobException e) {
      return e.getMessage();
    }
    // register job on server
    registerClusterJob(getCommandName(), bwaJob);
    // register result files ( saifile and stderr, as named in clusterservice_definition )
        for download
    String resultPrefix = command.getOptionValue(OPT_RESULT);
    registerClusterJobFile(getCommandName(), "saifile", resultPrefix+".sai", JobFileType.
        RESULT_FILE);
    registerClusterJobFile(getCommandName(), "stderr", resultPrefix+".log", JobFileType.
        RESULT_FILE);

    return null;
  }

}
```

Listing 5.3: PipelineJobFactory

```
/** Project: CommonBioJava */
...
public class PipelineJobFactory
{
  // bwa
  public static final int BWA_ALN = 360;
  ...
  public ClusterJobInterface getInstance(int job_task, Object object)
  {
    ...
    switch(job_task)
    {
      ...
      case BWA_ALN:
        new_job=createJob(BwaAlnCSCommand.CMD_NAME);
```

```
          break;
        ...
      }
      ...
    return new_job;
  }
  ...
}
```

5.3.2. Splicing

Splicings wire pipeline parameters, input -, and output files for a command. Therefore, the method 'splice' must be implemented. Several methods (e.g. addArgument, handOverArgument) facilitate the setting of command line parameters, whereas readJobsFromStep returns links to previously finished pipeline jobs which are used by ClusterClientHelper.createClusterOutputFileURLByName to provide a reference to the old job's result file on the server (e.g. for setting the result as input for the new tool).

Listing 5.4: Splicing

```
/** Project: IGB−TranscriptomePipeline */
...
public class BwaAlnSESplicing extends AbstractPreprocPipelineSplicing {
  ...
  /**
   * Parses pipeline sequence input files and starts parallel BwaAln commands.
   * Maps pipeline arguments to BwaAlnCSCommand arguments and registers commands.
   */
  @Override
  public Map<String, List<String>> splice(PipelineInterface pipeline) throws
      PipelineExecutionException {
    String [] inputfiles = getPipelineOptionValue(pipeline, PreprocPipeline.
        OPTL_INPUTFILES).split(",");
    switch (fqIs) {
      case FQRAW: // upload fastq files from client to server
        return rawInputAlignment(pipeline, inputfiles);
      case FQCONVERTED: // use already processed (converted) files on server
        return convertedInputAlignment(pipeline);
      ...
    }
    throw new PipelineExecutionException("Unknown input data type given.");
  }

  private Map<String, List<String>> rawInputAlignment(PipelineInterface pipeline,
      String[] inputfiles) {
    Map<String, List<String>> returnValueMap = new HashMap<String, List<String
        >>();
    // no conversion − take raw input
    for (String inputfile : inputfiles ) {
```

```
        List<String> arguments = initializeDefaultClusterArguments(pipeline, false);
        addArgument(arguments, BwaAlnCSCommand.OPT_INPUT, inputfile);
        String jobKey = setCommonArguments(pipeline, arguments, new File(inputfile).getName());
        returnValueMap.put(jobKey, arguments);
      }
      return returnValueMap;
    }

    private Map<String, List<String>> convertedInputAlignment(PipelineInterface pipeline) throws PipelineExecutionException {
      Map<String, List<String>> returnValueMap = new HashMap<String, List<String>>();
      // get input files from FastqConverterCSCommand
      Map<String, ClusterJobInterface> converterJobs = readJobsFromStep(pipeline, pipeline.getCommandPosition(FastqConverterCSCommand.CMD_NAME));

      for (String converterJobKey : converterJobs.keySet()) {
        List<String> arguments = initializeDefaultClusterArguments(pipeline, false);
        ClusterJobInterface job = converterJobs.get(converterJobKey);
        try {
          addArgument(arguments, BwaAlnCSCommand.OPT_INPUT, ClusterClientHelper.createClusterOutputFileURLByName("outputfile", job));
        } catch (ClusterJobException e) {
          throw new PipelineExecutionException(e);
        }

        String newJobKey = setCommonArguments(pipeline, arguments, extractCommandSpecificName(converterJobKey));
        returnValueMap.put(newJobKey, arguments);
      }
      return returnValueMap;
    }

    private String setCommonArguments(PipelineInterface pipeline, List<String> arguments, String inputfilename) {
      if (hasPipelineOption(pipeline, PreprocPipeline.OPTL_PREFIX))
        addArgument(arguments, BwaAlnCSCommand.OPT_GENOME, getPipelineOptionValue(pipeline, PreprocPipeline.OPTL_GENOME));

      // pass through arguments
      handOverArgument(arguments, BwaAlnCSCommand.OPT_MED, pipeline);
      handOverArgument(arguments, BwaAlnCSCommand.OPT_MGO, pipeline);
      ...
      // don't cleanup - this is done after the pipeline finished
      setNoCleanupArgument(arguments);

      // set output directory and outputnames
      addArgument(arguments, AbstractPipelineCommand.OUTPUTDIRECTORY_LONG,
```

```
        getOutputDirectory(getPipelineOptionValue(pipeline, AbstractPipelineCommand.OUTPUTDIRECTORY_LONG), inputfilename, pipeline));
    addArgument(arguments, BwaAlnCSCommand.OPT_RESULT, getFastxUtils().getPrefix(inputfilename));

    // register arguments
    // FIELD_DELIMITER are not allowed in ClusterClientHelper
    return inputfilename.replaceAll(ClusterClientHelper.FIELD_DELIMITER, "_");
  }

}
```

5.3.3. Pipeline

In order to integrate a new command line tool into SIMPLEX the classes ExomeBwaSEPipeline (SE data) and ExomeBwaPEPipeline (PE data) need to be updated. CSCommand parameters which should be available to the user must be added to the pipeline by extending the method getPipelineOptions. Parameter validity checks must be included in the method checkParameters. SetupPipelineImplementation defines the order of command line tools called on the server. This method must integrate the new CSCommand and its Splicing (see section 5.3.2) to call the application. Additionally an integer variable storing the application call's position within the pipeline must be created to be used in the method getCommandPosition.

Listing 5.5: Splicing

```
/** Project: IGB-TranscriptomePipeline */
 ...
public class ExomeBwaSEPipeline extends PreprocSEPipeline {
   ...
  private int bwaAlnPos = -1;
   ...
  @Override
  public Options getPipelineOptions() throws CommandIntegrityException {
     ...
    makeSubcommandOptionsPublic(pipelineOptions, new BwaAlnCSCommand(),
        new String[]{BwaAlnCSCommand.OPT_MED, BwaAlnCSCommand.OPT_MGO});
     ...
    return pipelineOptions;
  }

  @Override
  public String setupPipelineImplementation(CommandLine command, Options options) {
     ...
    try {
      // bwa aln
      addPipelineComponentAtPosition(pos, new BwaAlnSESplicing(isCs, dataType), new BwaAlnCSCommand());
      bwaAlnPos = pos++;
```

```
        ...
      }
        ...
    }

    @Override
    public int getCommandPosition(String cmdName) throws PipelineExecutionException {
      ...
      // bwa aln
      if (BwaAlnCSCommand.CMD_NAME.equalsIgnoreCase(cmdName)) {
        return bwaAlnPos;
      }
      ...
      return position;
    }

    @Override
    public String checkParameters(CommandLine command) {
      ...
      error = checkParameter(command, PreprocPipeline.OPTS_INPUTFILES);
      if (error != null)
        return error;
      ...
    }
  ...
}
```

5.4. Integrate new species

SIMPLEX was primarily designed to analyze human exome sequencing data. However, it is possible to extend SIMPLEX to include other species. Therefore, the following steps must be done:

5.4.1. Server side adaptations

1. Create the following folder structure on the server in folder */export/data/genomes*.

```
/export/data/genomes/
              |-- species_name/
                    |-- annotations/
                    |-- |-- annovar/
                    |-- |-- gatk/
                    |-- programs/
                          |-- |-- bwa/
                          |-- |-- gatk/
```

2. Prepare the reference genome:

a) On the server, change to directory */export/data/genomes/species_name/* and download the species' reference in fasta format. The whole reference must be stored in one file and be named as *species_name.fasta*.

b) Call picard's CreateSequenceDictionary to generate the fastq sequence dictionary file (see picards's man page and GATK's info page for further details). The resulting dictionary should be stored as *species_name.dict*.

c) Generate fasta index using samtools.

d) Create file *species_name.size* containing the number of bases in the reference genome in the format 'Size: #bases'.

e) Generate bwa reference index for nucleotide and color space in folder */export/data/genomes/species_name/programs/bwa/* as described in bwa's man page (use option -c to enable color-space indexing).

3. Prepare annotations for GATK tools:

   a) Change to directory */export/data/genomes/species_name/annotations/gatk/*.

   b) Download dbSNP and refSeq files and rename them to *annotation.dbSNP.txt* and *annotation.refSeq.txt*, respectively.

   c) For both databases, call GATK's *sortByRef.pl* to generate reference ordered files. The files must be named dbSNP.rod and refSeq.rod and must be stored in */export/data/genomes/species_name/programs/gatk/*.

4. Prepare annotations for annovar:

   a) Change to directory */export/data/genomes/species_name/annotations/annovar/*.

   b) Follow instructions on annovar's FAQ site on how to download and prepare files for using annovar to annotate other species.

5. Update */home/jclusterservice/jclusterservice/config/clusterresource_definition.properties* to include

```
# Picard
picard_124:species_name=/export/data/genomes/species_name/species_name.fasta
picard_124:species_name.color=/export/data/genomes/species_name/species_name.
    fasta

# BWA
bwa_0510_reference:species_name=/export/data/genomes/species_name/programs/bwa
    /species_name.ns.fasta
bwa_0510_reference:species_name.color=/export/data/genomes/species_name/programs
    /bwa/species_name.cs.fasta

#Fold coverage
foldcoverage-genomesizefile:species_name=/export/data/genomes/species_name/
    programs/gatk/latest/species_name.size
foldcoverage-genomesizefile:species_name.color=/export/data/genomes/species_name/
    programs/gatk/latest/species_name.size
```

```

#GATK
gatk_reference :species_name=/export/data/genomes/species_name/species_name.fasta
gatk_reference :species_name.color=/export/data/genomes/species_name/species_name.fasta

gatk_refseq :species_name=/export/data/genomes/species_name/programs/gatk/refSeq.pa.rod
gatk_refseq :species_name.color=/export/data/genomes/species_name/programs/gatk/refSeq.pa.rod

gatk_dbsnp:species_name=/export/data/genomes/species_name/programs/gatk/dbSNP.pa.rod
gatk_dbsnp:species_name.color=/export/data/genomes/species_name/programs/gatk/dbSNP.pa.rod

gatk_annotations:species_name=/export/data/genomes/species_name/annotations/gatk/
gatk_annotations:species_name.color=/export/data/genomes/species_name/annotations/gatk/

#Annovar
annovar_referencedb:species_name=/export/data/genomes/species_name/annotations/annovar/
```

5.4.2. Adaptations to the source code

1. Checkout SIMPLEX source code (subversion links are provided at http://simplex.i-med.ac.at).
2. Integrate new species to class *GenomeReferenceHelper* by updating methods *checkParameters* and *getGenomedefinition*.
3. Create class *SpeciesName* which implements interface *GenomeDefinition* (class *HG19Genome* can be used for guidance).
4. Rebuild exomePipeline.jar (ant task available exomeClpJar-Slim in file *TranscriptomePipeline/systemcommands/build.xml*), rename it to simplex.jar and update the client.

A. FAQ

A.1. Change password

Use the Linux command `passwd`

A.2. What can I do if simplex prints "Unable to connect to JClusterService."

- Configure the ip-address. If your network has a dhcp server you can use `sudo dhcp ethX`
- Stop and start JClusterservice `sudo service jclusterservice stop|start`

A.3. ToDo

- how to stop the cloud image
- link to Sun Grid Engine

Bibliography

[1] E Afgan, D Baker, N Coraor, B Chapman, A Nekrutenko, and J Taylor. Galaxy cloudman: delivering cloud compute clusters. *BMC Bioinformatics*, 11 Suppl 12:S4–S4, 2010.

[2] P J Cock, C J Fields, N Goto, M L Heuer, and P M Rice. The sanger fastq file format for sequences with quality scores, and the solexa/illumina fastq variants. *Nucleic Acids Res*, pages –, 2009.
