# Supplementary material for: SIMPLEX: Cloud-Enabled Pipeline for the Comprehensive Analysis of Exome Sequencing Data
Source: PLoS One. 2012 Aug 1;7(8):e41948. doi: 10.1371/journal.pone.0041948 (PMC3411592; doi:10.1371/journal.pone.0041948)
Supplement: Supplementary Material S2 — QA report after raw sequence preprocessing for sample SRR063831. (PDF) [file pone.0041948.s008.pdf]

Statistics on FASTQ Files for NGS Data

ICBI - Section for Bioinformatics

May 11, 2012

This document provides information about quality values and their occurrence based on the sequence file *SRR063831.fastq*.

1 Sequenced Read Lengths

Displaying basic read length information from file *SRR063831_readlengths.txt*.

- number of reads analyzed

```
> read_length
```

```
[1] 22.634.594
```

- statistics

```
> summary(read_lens)
```

```
   Min. 1st Qu.  Median    Mean 3rd Qu.    Max.
  25.00   76.00   76.00   75.97   76.00   76.00
```

- histogram

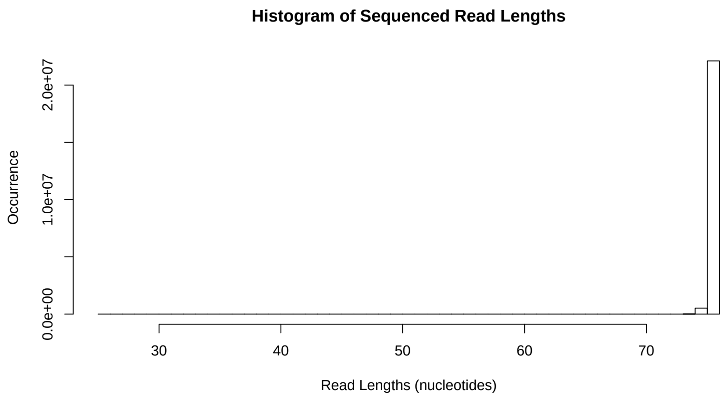

Supplementary Figure 1: Histogram of sequenced read lengths.

- boxplot

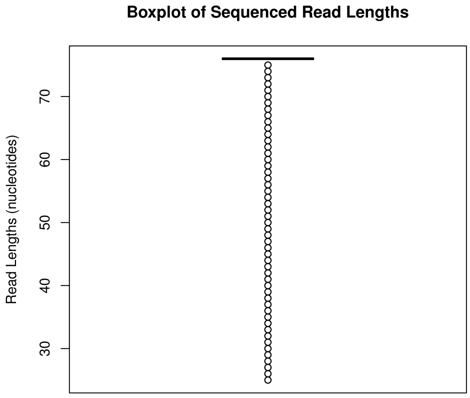

Supplementary Figure 2: Boxplot of sequenced read lengths.

2 Base Call Comparison

2.1 Comparison of Base Calls according to their Read Position

- quality value medians

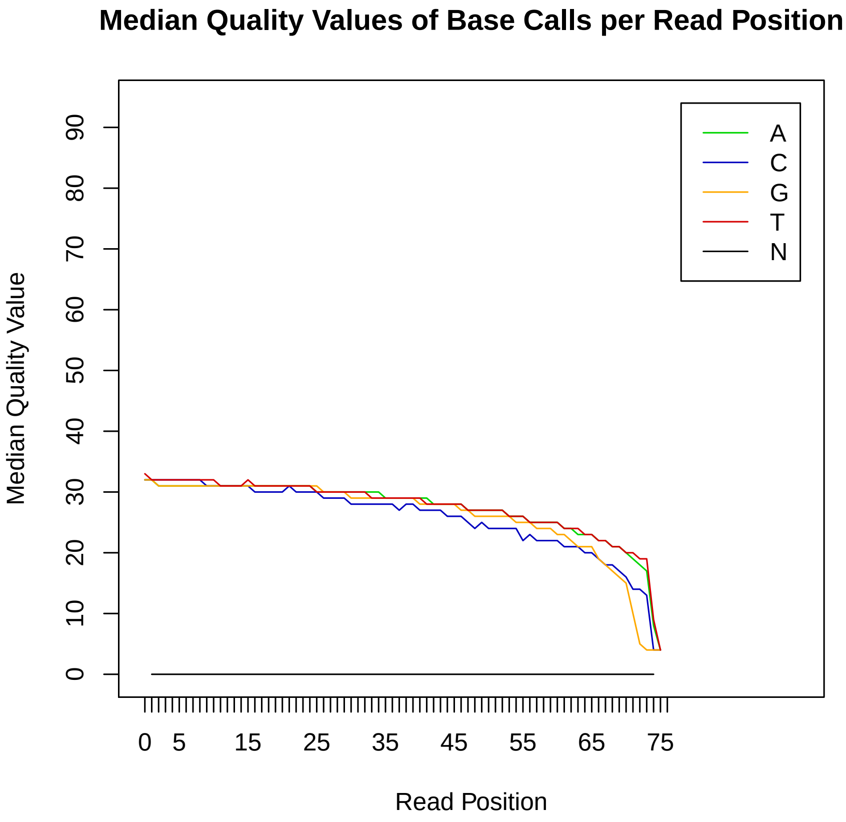

Supplementary Figure 3: Meadian quality values of each base per read position. Missing points in the graphs may occur due to no detection of the base at this certain position.

- absolute quality value frequencies

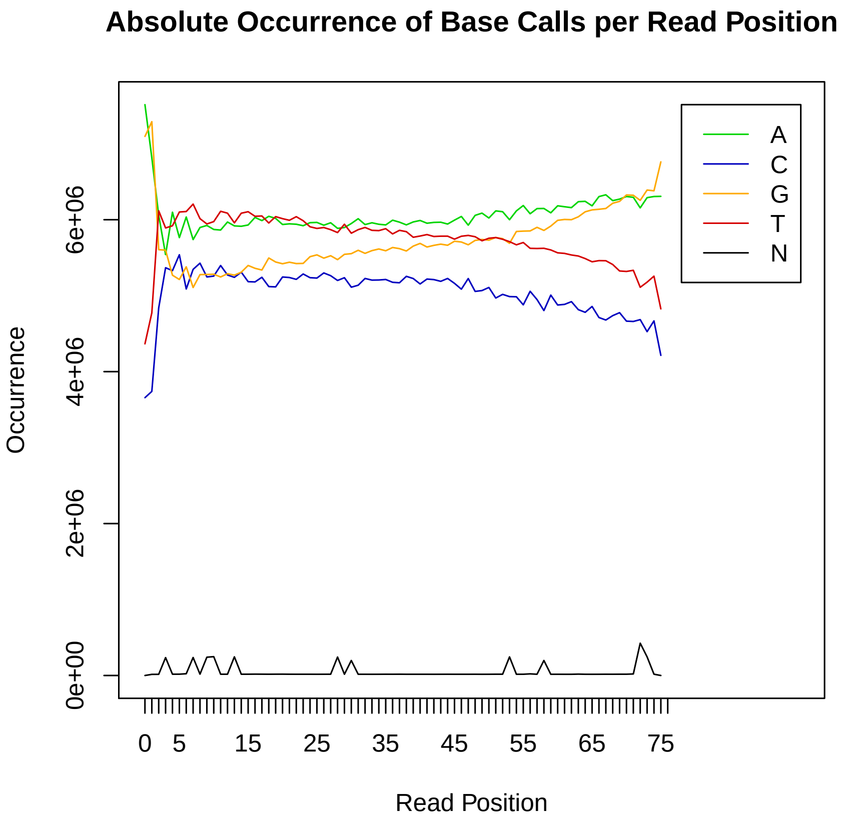

Supplementary Figure 4: Absolute occurrence of base calls per read position.

- contents in %

```
> GC_content

[1] 47.62411

> AT_content

[1] 52.15411

> N_content

[1] 0.2217846
```

2.2 Distribution of Occurrences of IUPAC Code N (Gaps) per Read

- number of reads containing no gaps

  > *no_n_per_read*

  `[1] 20.222.826`

- number of reads consisting only of gaps

  > *all_n_per_read*

  `[1] 0`

- number of reads containing at least one gap and at least one nucleotide

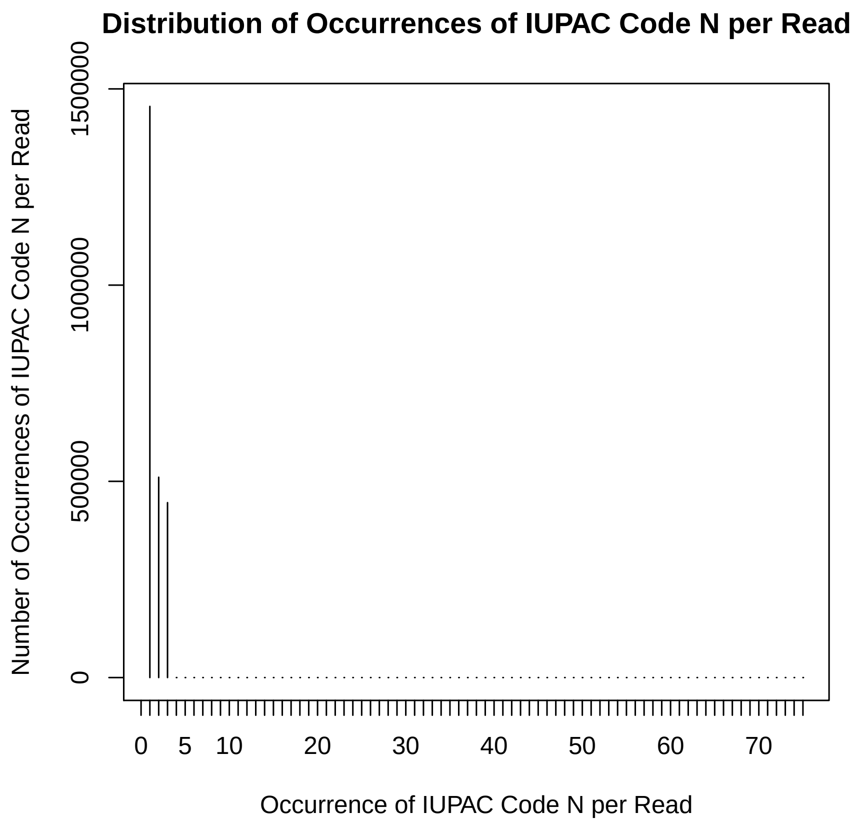

Supplementary Figure 5: Number of reads with at least one gap and one nucleotide

3 Quality Values for IUPAC Nucleotide Code A

Showing information about quality values for Adenine obtained from file *SRR063831_-A_qualities.txt*

- normalized heatmap of quality value distribution per read position. Normalization is done by division by the value's colsum.

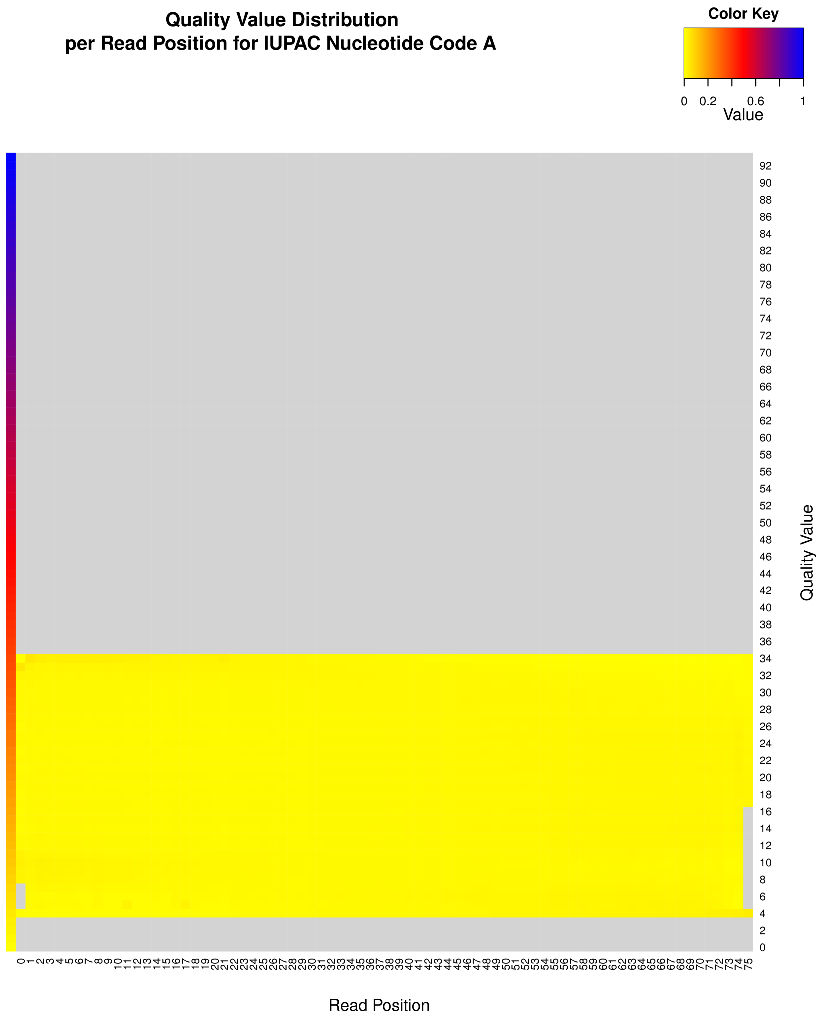

Supplementary Figure 6: Heatmap showing the quality value distribution per read position for Adenine. The color key on the upper right side and on the vertical left stripe encode the values $\in]0;1]$. Lightgrey areas mark quality values that did not occur. For displaying reasons only every other quality value is labeled.

4 Quality Values for IUPAC Nucleotide Code C

Showing information about quality values for Cytosine obtained from file *SRR063831_-C_qualities.txt*

- normalized heatmap of quality value distribution per read position. Normalization is done by division by the value's colsum.

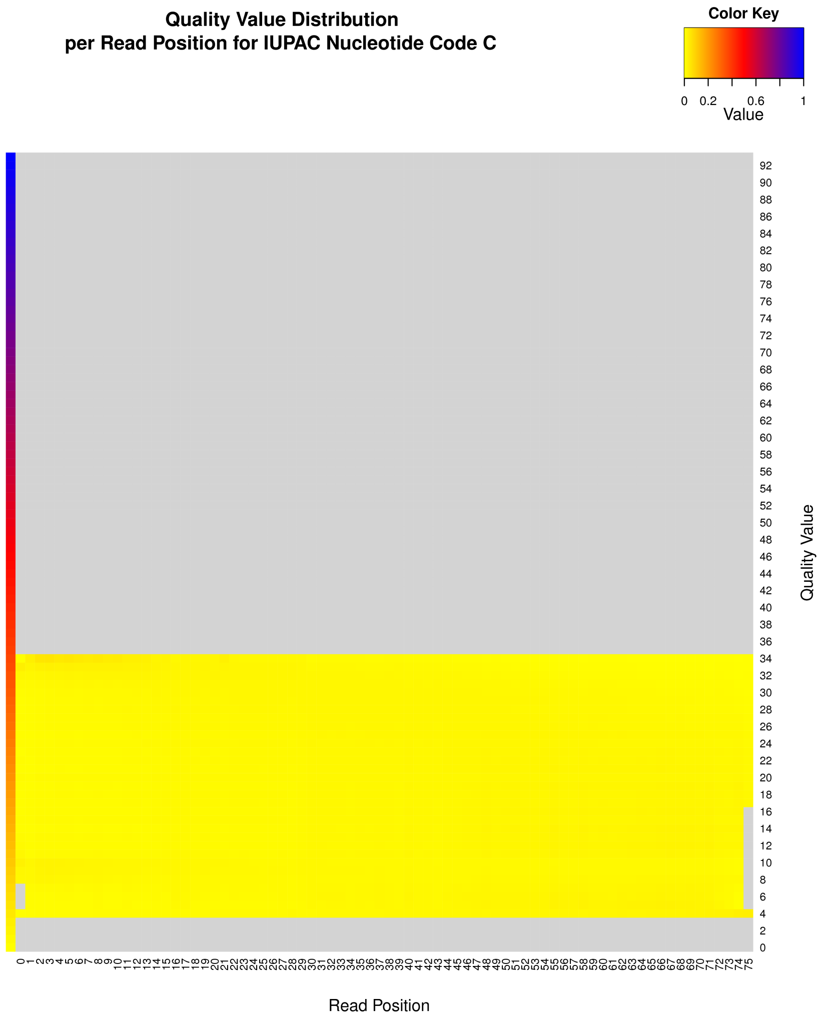

Supplementary Figure 7: Heatmap showing the quality value distribution per read position for Cytosine. The color key on the upper right side and on the vertical left stripe encode the values $\in]0;1]$. Lightgrey areas mark quality values that did not occur. For displaying reasons only every other quality value is labeled.

5 Quality Values for IUPAC Nucleotide Code G

Showing information about quality values for Guanine obtained from file *SRR063831_-G_qualities.txt*

- normalized heatmap of quality value distribution per read position. Normalization is done by division by the value's colsum.

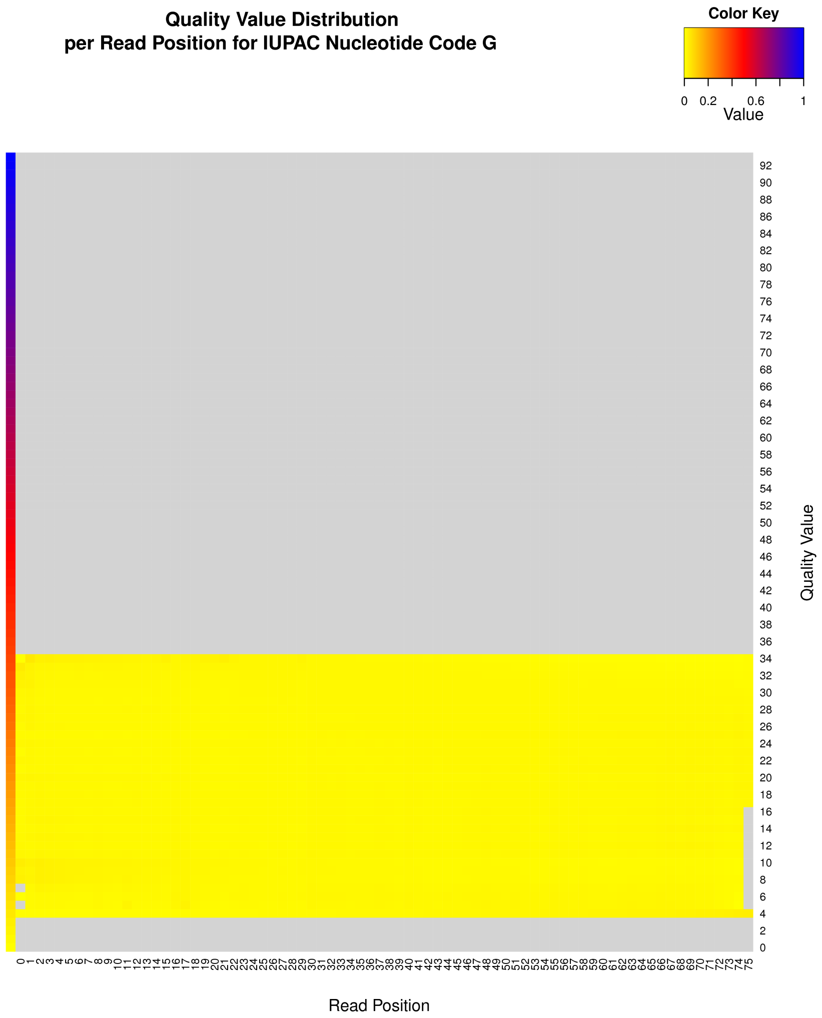

Supplementary Figure 8: Heatmap showing the quality value distribution per read position for Guanine. The color key on the upper right side and on the vertical left stripe encode the values ∈]0; 1]. Lightgrey areas mark quality values that did not occur. For displaying reasons only every other quality value is labeled.

6 Quality Values for IUPAC Nucleotide Code T

Showing information about quality values for Thymine obtained from file *SRR063831_-T_qualities.txt*

- normalized heatmap of quality value distribution per read position. Normalization is done by division by the value's colsum.

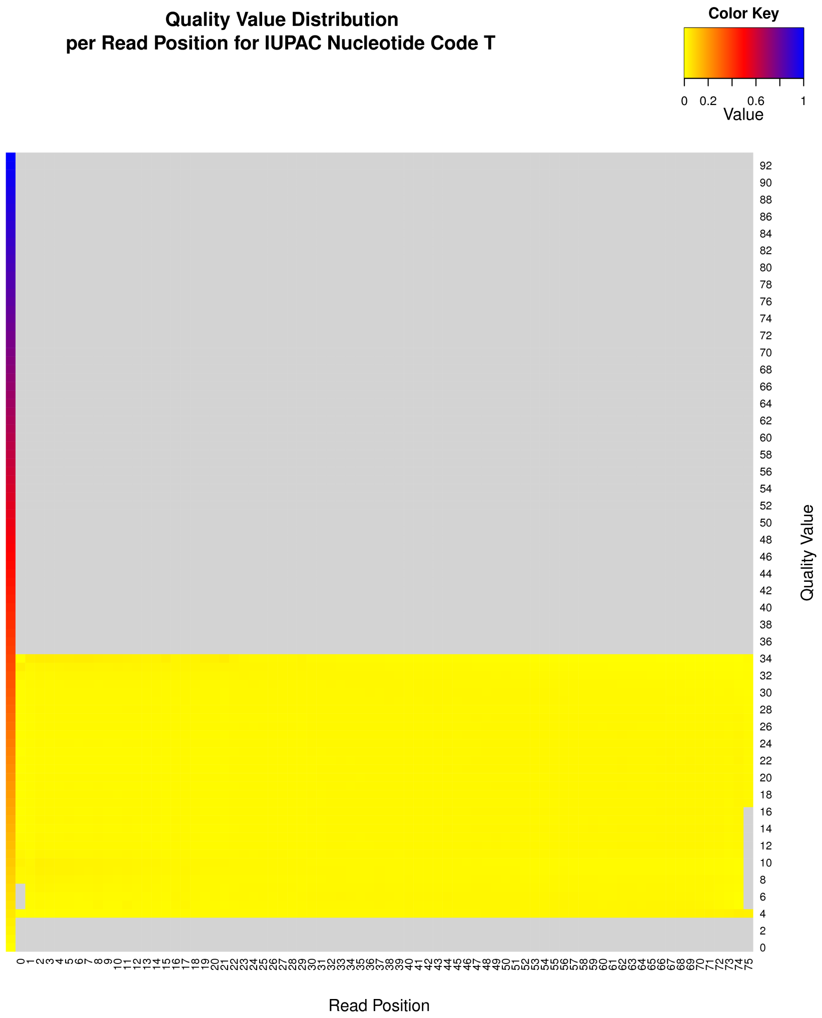

Supplementary Figure 9: Heatmap showing the quality value distribution per read position for Thymine. The color key on the upper right side and on the vertical left stripe encode the values $\in]0;1]$. Lightgrey areas mark quality values that did not occur. For displaying reasons only every other quality value is labeled.

7 Quality Values for IUPAC Nucleotide Code N

Showing information about quality values for not identified bases obtained from file *SRR063831_N_qualities.txt*

- normalized heatmap of quality value distribution per read position. Normalization is done by division by the value's colsum.

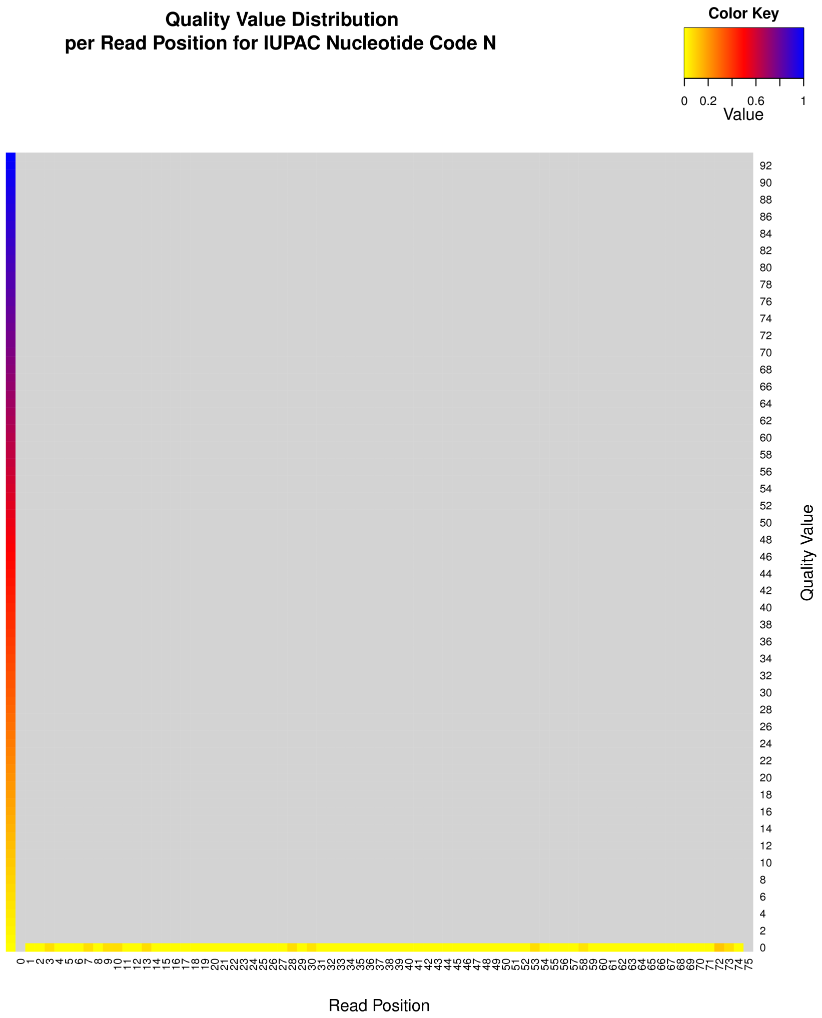

Supplementary Figure 10: Heatmap showing the quality value distribution per read position for not identified bases. The color key on the upper right side and on the vertical left stripe encode the values $\in]0;1]$. Lightgrey areas mark quality values that did not occur. For displaying reasons only every other quality value is labeled.
